# Mechanism of aminoacyl-tRNA acetylation by an aminoacyl-tRNA acetyltransferase AtaT from enterohemorrhagic *E. coli*

**DOI:** 10.1038/s41467-020-19281-z

**Published:** 2020-10-28

**Authors:** Yuka Yashiro, Yuriko Sakaguchi, Tsutomu Suzuki, Kozo Tomita

**Affiliations:** 1 Department of Computational Biology and Medical Sciences, Graduate School of Frontier Sciences, The University of Tokyo, Kashiwa, Chiba 277-8562 Japan; 2 Department of Chemistry and Biotechnology, Graduate School of Engineering, The University of Tokyo, Bunkyo-ku, Tokyo 113-8656 Japan

**Keywords:** Bacteria, tRNAs, X-ray crystallography

## Abstract

Toxin-antitoxin systems in bacteria contribute to stress adaptation, dormancy, and persistence. AtaT, a type-II toxin in enterohemorrhagic *E. coli*, reportedly acetylates the α-amino group of the aminoacyl-moiety of initiator Met-tRNAf^Met^, thus inhibiting translation initiation. Here, we show that AtaT has a broader specificity for aminoacyl-tRNAs than initially claimed. AtaT efficiently acetylates Gly-tRNA^Gly^, Trp-tRNA^Trp^, Tyr-tRNA^Tyr^ and Phe-tRNA^Phe^ isoacceptors, in addition to Met-tRNAf^Met^, and inhibits global translation. AtaT interacts with the acceptor stem of tRNAf^Met^, and the consecutive G-C pairs in the bottom-half of the acceptor stem are required for acetylation. Consistently, tRNA^Gly^, tRNA^Trp^, tRNA^Tyr^ and tRNA^Phe^ also possess consecutive G-C base-pairs in the bottom halves of their acceptor stems. Furthermore, misaminoacylated valyl-tRNAf^Met^ and isoleucyl-tRNAf^Met^ are not acetylated by AtaT. Therefore, the substrate selection by AtaT is governed by the specific acceptor stem sequence and the properties of the aminoacyl-moiety of aminoacyl-tRNAs.

## Abstract (web summary)

AtaT is a type-II toxin from enterohemorrhagic *E. coli*, reported to acetylate the aminoacyl-moiety of initiator Met-tRNAf^Met^, thus inhibiting translation initiation. Biochemical analysis suggests that AtaT has a broader specificity for aminoacyl-tRNAs and inhibits global translation. Structure of AtaT in complex with acetylated Met-tRNAf^Met^ offers insight into the substrate selection by the enzyme.

## Introduction

A bacterial toxin–antitoxin (TA) module is a gene pair of a protein toxin that induces cell growth arrest and an antitoxin that counteracts the toxin^1–4^. Toxins in TA-modules target pivotal cellular processes, including DNA replication, transcription, translation, and cell wall synthesis^3^. TA modules have been implicated in bacterial stress adaptation, persistence, and dormancy to survive under environmental stresses, and are involved in bacterial pathogenesis^5–8^. Based on the nature of antitoxins and the mechanism by which they neutralize the toxin activity, TA modules are classified into six different types^9^. In the type II TA system, under normal physiological conditions, the protein antitoxin inhibits the activity of the toxin by direct protein–protein interactions. Since the type II antitoxins are susceptible to proteolytic degradation due to their inherently disordered regions, under stress conditions, antitoxin degradation is induced by proteases, such as Lon and ClpP, thus leading the activation of the toxins^10,11^.

During the last few years, a new class of type II toxins belonging to the Gcn5-related *N*-acetyltransferase (GNAT) family has been identified^12–17^. GNAT toxins acetylate the α-amino group of the aminoacyl-moiety of aminoacyl-tRNAs, using acetyl CoA (Ac-CoA) as the acetyl group donor, and inhibit cellular translation. The GNAT toxins reportedly have different substrate specificities for their target aminoacyl-tRNAs. TacT, TacT2, and TacT3 in *Salmonella* can acetylate several isoaccepting aminoacyl tRNAs^12,18^. TacT acetylates various aminoacyl-tRNA isoacceptors for glycine, isoleucine/leucine, tryptophan, and serine in vitro and may inhibit global cellular translation^18^. The TacT activity contributes to intracellular persistence in macrophages^12,18^. ItaT, identified in the *E. coli* HS strain, reportedly acetylates the isoaccepting aminoacyl tRNAs for isoleucine, valine, and methionine in vivo and in vitro, and may inhibit global cellular translation^15,19^. On the other hand, AtaT of the enterohemorrhagic *E. coli* O157:H7 strain specifically acetylates the initiator methionyl-tRNAf^Met^ (Met-tRNAf^Met^) in vitro^13^. AtaT was assumed to block the translation initiation step, because acetylated Met-tRNAf^Met^ cannot interact with translation initiation factor 2 complexed with GTP (IF2-GTP). GNAT toxins have also been identified in *Klebsiella pneumoniae* (KacT)^14,17^ and the *Shigella flexeneri* pINV plasmid (GmvT)^16^, although their aminoacyl-tRNA targets remain unknown.

Recently, the crystal structures of AtaT and its complex with the cognate antitoxin AtaR have been reported^20,21^. The structural and biochemical studies revealed that the homodimerization of AtaT is required for both the enzymatic activity and the toxicity. The electrostatic surface potential of the dimeric AtaT showed that a positively charged patch is created upon dimer formation, implying the requirement of dimer formation for aminoacyl-tRNA binding^21^. However, the molecular mechanism for specific aminoacyl-tRNA recognition by AtaT has remained elusive.

Based on structural and biochemical studies, we now show that AtaT interacts with the acceptor stem region of the aminoacyl-tRNA, and the G–C pairs at the bottom half of the acceptor stem are important for acetylation. Consistent with this finding, AtaT also acetylates several other aminoacyl-tRNAs possessing G–C pairs at the bottom half of the acceptor stem in vivo and in vitro. AtaT also recognizes the properties of the side-chain of the aminoacyl-moiety of the substrate aminoacyl-tRNA for acetylation. Thus, AtaT has broader specificity toward aminoacyl-tRNAs, and the selection of substrate aminoacyl-tRNAs is governed by both the specific sequence in the acceptor stem and the side chain of the aminoacyl-moiety of aminoacyl-tRNAs.

## Results

### AtaT recognizes both tRNA sequence and aminoacyl moiety

**Fig. 1:**
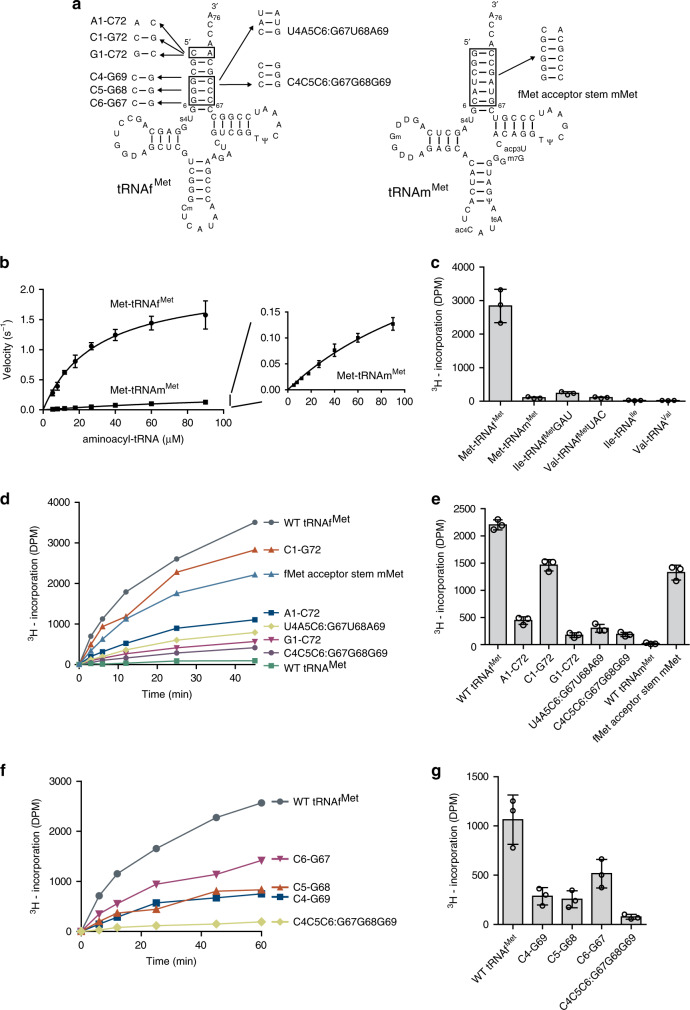
Elements in Met-tRNAf^Met^ required for acetylation by AtaT. **a** Secondary structures of *E. coli* initiator tRNAf^Met^ and elongator tRNAm^Met^ in cloverleaf forms. The nucleotide substitutions in the tRNAf^Met^ and tRNAm^Met^ transcripts used for assays in **d** and **f** are shown by boxes and arrows. **b** Steady-state kinetics of methionyl-tRNAf^Met^ (Met-tRNAf^Met^) and methionyl-tRNAm^Met^ (Met-tRNAm^Met^) acetylation by AtaT. The initial velocities of acetylation of Met-tRNAf^Met^ and Met-tRNAm^Met^ were measured with various concentrations of Met-tRNAf^Met^ and Met-tRNAm^Met^ (5–90 μM). Magnified view of the graph for the acetylation of Met-tRNAm^Met^. tRNAf^Met^ and tRNAm^Met^ were overexpressed in *E. coli* and purified. **c** In vitro acetylation of misaminoacylated Val-tRNAf^Met^UAC and Ile-tRNAf^Met^GAU. tRNAf^Met^UAC and tRNAf^Met^GAU were overexpressed in *E. coli*, purified and charged with valine and isoleucine by valyl-tRNA and isoleucyl-tRNA synthetases, respectively. Aminoacyl-tRNAs (10 μM) were used for the acetylation by AtaT and incubated for 12 min at 37 °C. **d**, **e** In vitro acetylation of Met-tRNAm^Met^, Met-tRNAf^Met^, and its variants shown in **a**. tRNAm^Met^, tRNAf^Met^, and its variants are in vitro transcripts prepared by T7 RNA polymerase. **d** Time courses of the acetylation of Met-tRNAf^Met^, Met-tRNAm^Met^, and its variants in **a**. **e** Quantification of the acetylation efficiencies in **d**. The initial velocities of the acetylation of Met-tRNAf^Met^, Met-tRNAm^Met^, and its variants were measured. **f** Time courses of the acetylation of Met-tRNAf^Met^ variants with mutations in the bottom half of the three consecutive G–C pairs in **a**. **g** Quantification of the acetylation efficiencies in **f**, as in **e**. The bars in the graph are SD of three independent (*n* = 3) experiments, and the data are presented as mean values ± SD.

AtaT was previously shown to acetylate initiator methionyl-tRNAf^Met^ (Met-tRNAf^Met^) specifically, but not the elongator methionyl-tRNAm^Met^ (Met-tRNAm^Met^) in vitro^13^. Consistent with the previous report, the steady-state kinetics of Met-tRNAf^Met^ and Met-tRNAm^Met^ acetylation by AtaT showed different acetylation efficiencies between Met-tRNAf^Met^ and Met-tRNAm^Met^ (Fig. 1a, b). While the *K*_m_ value of Met-tRNAf^Met^ is ~29.6 ± 3.8 μM, the *K*_m_ value of Met-tRNAm^Met^ is 172 ± 43 μM. The *V*_max_ values of Met-tRNAf^Met^ and Met-tRNAm^Met^ acetylation were estimated to be 2.1 ± 0.12 and 0.38 ± 0.07 s^−1^, respectively. The overall reaction efficiency of Met-tRNAf^Met^ was ~30-fold higher than that of Met-tRNAm^Met^, and thus Met-tRNAf^Met^ is a much better substrate than Met-tRNAm^Met^ for AtaT in vitro. Since both Met-tRNAf^Met^ and Met-tRNAm^Met^ are charged with methionine, this result suggests that AtaT discriminates the tRNA sequence of Met-tRNAf^Met^ from that of Met-tRNAm^Met^.

To evaluate the recognition of the aminoacyl moiety of Met-tRNAf^Met^ by AtaT, mutant tRNAf^Met^ molecules with a UAC anticodon (tRNAf^Met^UAC) and a GAU anticodon (tRNAf^Met^GAU) were prepared and charged with valine and isoleucine by valyl-tRNA synthase and isoleucyl-tRNA synthetase, respectively^22,23^. The acetylations of valyl-tRNAf^Met^UAC (Val-tRNAf^Met^UAC) and isoleucyl-tRNAf^Met^GAU (Ile-tRNAf^Met^GAU) by AtaT were tested in vitro. Although Val-tRNAf^Met^UAC and Ile-tRNAf^Met^GAU have the same nucleotide sequence as Met-tRNAf^Met^, except for the anticodon sequence, they were not acetylated by AtaT in vitro (Fig. 1c). Since AtaT recognizes the acceptor region of tRNAf^Met^, as described below, these results suggest that both the nucleotide sequence of tRNA and the side chain of the aminoacyl moiety of aminoacyl-tRNAs are important for the acetylation of aminoacyl-tRNAs by AtaT.

### AtaT recognizes the acceptor region of tRNA

To identify the recognition elements in tRNAf^Met^ that allow AtaT to discriminate tRNAf^Met^ from tRNAm^Met^, we first generated a chimeric tRNAm^Met^ transcript with the acceptor stem sequence of tRNAf^Met^ (fMet acceptor stem mMet) (Fig. 1a). Replacing the tRNAm^Met^ acceptor stem with the tRNAf^Met^ acceptor stem conferred efficient acetylation of the mutant chimeric tRNAm^Met^ transcript, to almost 60% of that of the wild-type tRNAf^Met^ transcript (Fig. 1d, e), while the Met-tRNAm^Met^ transcript was not acetylated under these conditions. This result suggests that the elements recognized by AtaT reside in the acceptor stem region of tRNAf^Met^.

The sequence differences in the acceptor stems between tRNAf^Met^ and tRNAm^Met^ are located at the top of the acceptor helix and the bottom half of the stem (Fig. 1a). The C1–A72 mispair at the top of the acceptor helix is one of the characteristic features of tRNAf^Met^. Mutations of C1–A72 to the A1–C72 or G1–C72 base pair of the tRNAf^Met^ transcript decreased the acetylation efficiency to about 20% or 10% of that of the wild-type Met-tRNAf^Met^, respectively, while the mutation of C1–A72 to the C1–G72 base-pair decreased the acetylation efficiency to about 65% of the wild-type Met-tRNAf^Met^ (Fig. 1d, e). Thus, the mispair at the top of the acceptor helix is not necessarily required for the acetylation of Met-tRNAf^Met^ by AtaT, but the nucleotide compositions at positions 1 and 72, in the context of the nucleotide sequences in the acceptor helix, are important for acetylation by AtaT, as described below. On the other hand, the mutation of the consecutive three G–C pairs, G4G5G6:C67C68C69, in the tRNAf^Met^ transcript to the corresponding sequence of tRNAm^Met^ (U4A5C6:G67U68A69) decreased the acetylation efficiency to about 10% of that of the wild-type Met-tRNAf^Met^ (Fig. 1d, e). Reversing the three consecutive G–C base pairs in the tRNAf^Met^ transcript to the three consecutive C–G pairs, C4C5C6:G67G68G69, reduced the acetylation efficiency to about 10% of that of wild-type Met-tRNAf^Met^ (Fig. 1d, e). The mutations of one of the three consecutive G–C pairs in the tRNAf^Met^ transcript to a C–G pair (C4–G69, C5–G68, or C6–G67) decreased the acetylation efficiency to 25–50% of that of the wild-type Met-tRNAf^Met^ (Fig. 1f, g). These results suggest that AtaT mainly recognizes the acceptor region of tRNAf^Met^ for discrimination from tRNAm^Met^. In particular, the bottom-half consecutive three G–C base-pairs in the tRNAf^Met^ acceptor stem, rather than the C1–A72 mispair, are more important for the acetylation of Met-tRNAf^Met^ by AtaT.

### Structural determination of AtaT–Ac–Met–tRNAf^Met^ complex

To uncover the molecular basis of Met-tRNAf^Met^ recognition by AtaT, AtaT, and acetyl–Met–tRNAf^Met^ (Ac–Met–tRNAf^Met^) were co-crystallized and the structure was determined. Since Ac–Met–tRNAf^Met^ is chemically more stable than Met–tRNAf^Met^, Ac–Met–tRNAf^Met^ was used instead of Met–tRNAf^Met^ for crystallization. The structure should represent a post-reaction stage in which the acetyl group of acetyl-CoA (Ac-CoA) is transferred to the α-amino group of the aminoacyl moiety of Met–tRNAf^Met^, and the CoA byproduct is released from the AtaT catalytic site.

The crystal belongs to the space group *P2*_*1*_*2*_*1*_*2* and contains eight AtaT molecules and two Ac–Met–tRNAf^Met^ molecules in the asymmetric unit. The initial phase was determined by the molecular replacement method, using the AtaT homodimer (PDB: 6GTP)^20^ and tRNAf^Met^ structures in the structure of the complex of Met–tRNAf^Met^ formyltransferase and formyl–Met–tRNAf^Met^ (PDB: 2FMT)^24^ as search models. The structure was model-built and refined to an *R* factor of 28.6% (*R*_free_ = 34.1%) at 3.8 Å resolution. Detailed crystallographic data collection and refinement statistics are provided in Table 1.

**Table 1:** Data collection and refinement statistics.

	AtaT–Ac–Met–tRNAf^Met^
*Data collection*	
Space group	*P2*_1_*2*_1_*2*
Cell dimensions	
* a*, *b*, *c* (Å)	269.83, 68.28, 136.18
Wavelength (Å)	0.98000
Resolution (Å)^a^	48.21–3.804 (3.94–3.804)
*R*_sym_^a^	0.449 (3.45)
*I*/*σI*^a^	8.4 (1.3)
CC_1/2_^a^	0.999 (0.508)
Completeness (%)^a^	99.64 (99.27)
Redundancy^a^	26.2 (26.6)
*Refinement*	
Resolution (Å)	48.21–3.804 (3.94–3.804)
No. of reflections	25,488
*R*_work_/*R*_free_ (%)	28.60/34.06
No. of atoms	
Protein	10,968
tRNA	3206
*B*-factors (Å^2^)	
Protein	142.23
tRNA	131.99
R.m.s. deviations	
Bond lengths (Å)	0.005
Bond angles (°)	1.09

^a^Values in parentheses are for the highest-resolution shell.

Of the eight AtaT molecules in the asymmetric unit (AtaT1–8), six (AtaT3–8) formed three dimers (AtaT3–AtaT4, AtaT5–AtaT6, and AtaT7–AtaT8), and AtaT1 and AtaT2 each formed AtaT1–AtaT1′ and AtaT2–AtaT2′ dimers, respectively, through the crystallographic two-fold rotation axis (Supplementary Fig. 1). The 3′ single-stranded regions of the two tRNA molecules, tRNAf^Met^1 and tRNAf^Met^1′, and tRNAf^Met^2 and tRNAf^Met^2′ interact with the regions proximal to the two catalytic sites of AtaT1–AtaT1′ and AtaT2–AtaT2, respectively. Although AtaT molecules 3–8 also contact tRNA molecules, the contact sites are distant from the 3′ ends of the tRNAs. This indicates that the interactions between AtaT3–8 and tRNAs are non-physiological crystal-packing interactions. Hereafter, we focus on AtaT1–AtaT1′ complexed with tRNAf^Met^1 and tRNAf^Met^1′, and describe the interactions between AtaT and tRNA, and AtaT1, AtaT1′, tRNAf^Met^1 and tRNAf^Met^1′ as AtaTa, AtaTb, tRNAf^Met^a, and tRNAf^Met^b, respectively.

### Interaction of tRNA with AtaT homodimer

**Fig. 2:**
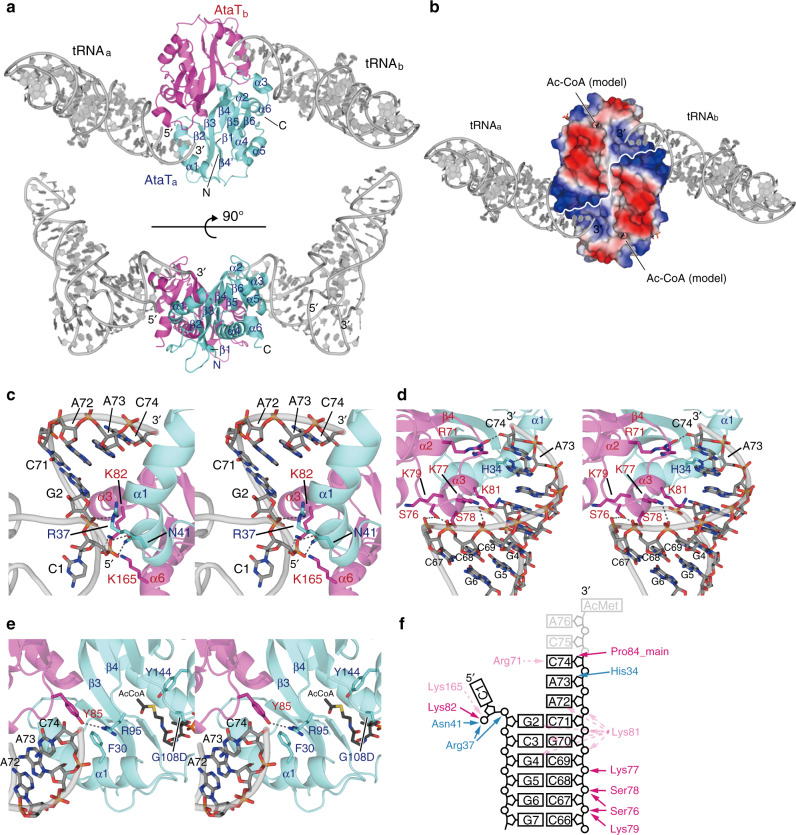
Structure of AtaT complexed with Ac–Met–tRNAf^Met^. **a** Overall structure of AtaT complexed with acetyl–Met–tRNAf^Met^ (Ac–Met–tRNAf^Met^). The ribbon models of the AtaTa, AtaTb, and Ac–Met–tRNAf^Met^ molecules (tRNAa and tRNAb) are colored cyan, magenta, and gray, respectively. **b** Electrostatic surface area potentials of the AtaT dimer in the complex with Ac–Met–tRNAf^Met^. Positively and negatively charged areas are colored blue and red, respectively. Acetyl-CoA was modeled in the active site of AtaT. **c**–**e** Stereoviews of detailed interactions between AtaT and Ac–Met–tRNAf^Met^. AtaTa and AtaTb are colored as in **a**. tRNAf^Met^ is shown as a gray stick model. Acetyl-CoA was modeled in the active site of AtaT in **e** as a black stick model. **f** Schematic representation of the interactions between AtaT and Ac–Met–tRNAf^Met^. Residues in AtaTa and AtaTb are colored cyan and magenta, respectively, as in **a**. All structural figures were prepared using PyMol (http://www.pymol.org).

In the structure of AtaT complexed with Ac–Met–tRNAf^Met^, two tRNAf^Met^ molecules (tRNAf^Met^a and tRNAf^Met^b) interact with an AtaT homodimer (AtaTa and AtaTb) (Fig. 2a). The 3′-terminal single-stranded region and acceptor stem of each tRNAf^Met^ interact with the positively charged interface between the two subunits of the AtaT dimer. The 3′-single-stranded termini of tRNAf^Met^a and tRNAf^Met^b are directed toward the active sites of AtaTa and AtaTb in the dimer, respectively (Fig. 2b). The binding of the acceptor stem region to the interface between the AtaT dimer subunits explains the previous results showing that the AtaT homodimerization is required for both the enzymatic and toxic activities of AtaT^21^. The structure also revealed that the recognition elements in tRNAf^Met^ for acetylation by AtaT reside in its acceptor stem region (Fig. 1d, e).

In the complex structure, the α3 in AtaTb penetrates the major groove of the acceptor helix of tRNAf^Met^a, and the α1 in AtaTa also penetrates the groove from another angle (Fig. 2c). As a result, C1–A72 at the top of the acceptor helix of tRNAf^Met^ are completely separated and the 5′-terminal C1 nucleoside is flipped. The 5′-phosphate of C1 interacts with K82 and K165 in AtaTb and with N41 and R37 in AtaTa (Fig. 2c, f). On the other hand, although 3′-C75A76–Ac–Met of Ac–Met–tRNAf^Met^ were not visible in the present structure (Supplementary Fig. 2), the nucleobases of A72, A73, and C73 are continuously stacked and the 3′-end is directed toward the catalytic site, where G108 and Y114 participate in Ac-CoA binding and catalysis, respectively^20,21^. The loop between α3 and β4 in AtaTb and α1 in AtaTa form the path for the 3′-end of tRNA to enter the catalytic site and interact with the 3′-single-stranded region of tRNAf^Met^ (Fig. 2d). The ribose 2′-OH groups of C74 and A73 in tRNAf^Met^ interact with the main-chain carbonyl oxygens of P84 in AtaTb and H34 in AtaTa, respectively, and the O2 atom of the C74 nucleobase of tRNAf^Met^ interacts with R71 in AtaTb. Residues S76, K77, S78, and K79 in α3 of AtaTb interact with the phosphate backbones of C67C68C69, at the bottom half of the acceptor helix. K81 in α3 is positioned so its side chain can interact with the phosphate backbones and nucleobases of consecutive G–C pairs, at the top-half of the acceptor helix (Fig. 2d, f). Although an in vitro analysis demonstrated the importance of the (G4G5G6:C67C68C69) sequence at the bottom half of the acceptor helix (Fig. 1d, e), no base-specific interactions were observed in the present structure, as described below.

The 3′-C75A76–Ac–Met region of Ac–Met–tRNAf^Met^ was disordered and not visible in the present structure. Y85 in the loop between α3 and β4 in AtaTb, and R95, which stacks with F30 in α1, in β4 of AtaTa form a hydrogen-bond, and these interactions would block the access of the 3′-end of Ac–Met–tRNAf^Met^ to the catalytic site (Fig. 2e). No significant structural differences were detected in a comparison of the catalytic pocket structure of AtaT complexed with Ac–Met–tRNAf^Met^ with that of *apo* AtaT (Supplementary Fig. 3). Thus, the present structure represents a post-reaction stage of Met–tRNAf^Met^ acetylation, where the Ac–Met–tRNAf^Met^ product is ready for release from the enzyme after the completion of the reaction. This would explain why no specific interaction between α3 and the bottom half of the acceptor helix was observed in the present structure. During the reaction stage of the acetylation of Met–tRNAf^Met^, the basic residues in α3 would specifically recognize the sequence and structure at the bottom half of the tRNAf^Met^ acceptor helix.

**Fig. 3:**
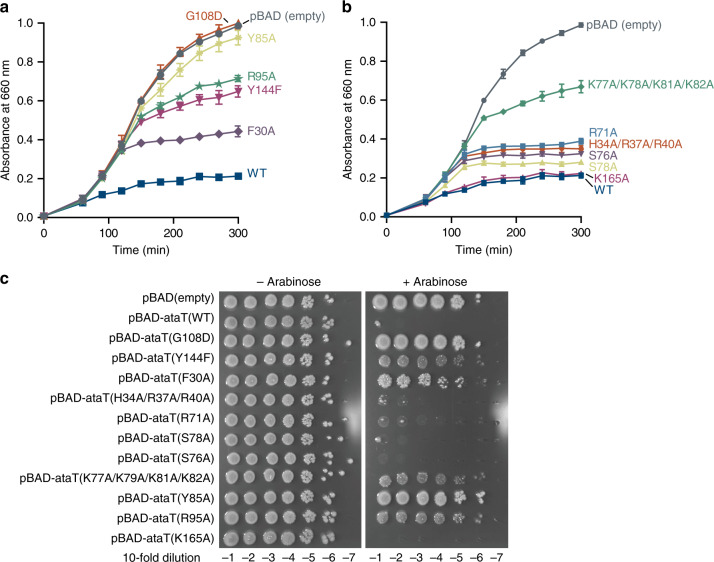
Toxicity of AtaT variants. **a**, **b** Growth curves of *E. coli* MG1655 transformed with pBAD-ataT and its variants. *E. coli* MG1655 transformed by pBAD-ataT or its variants was inoculated in LB containing 50 μg mL^−1^ ampicillin and 0.2% (w/v) arabinose at 37 °C. Bars in the graphs indicate the SD of three independent experiments. **c** Overnight cultures of *E. coli* MG1655 transformed with pBAD-ataT and its variants were serially diluted, and the dilutions were spotted on LB agar plates containing 50 μg mL^−1^ ampicillin and supplemented with 0.2% (w/v) arabinose or without arabinose. The plates were incubated at 37 °C overnight. The bars in the graph are SD of three independent (*n* = 3) experiments, and the data are presented as mean values ± SD.

To verify the effects of the above-mentioned residues on the activity of AtaT, AtaT mutants were assessed for the ability to inhibit *E. coli* cell growth (Fig. 3). G108D and Y144F mutants were used as positive controls, since the mutation of G108 to D108 causes AtaT to lose its toxicity by blocking Ac-CoA binding^20,21^, and the mutation of the catalytic residue Y144 to F144 also reduces AtaT toxicity in vivo^20,21,25^. As expected, the substitution of positively charged residues in helix α1 (H34A/R37A/R40A) attenuated the cell growth inhibition by AtaT. R71A slightly decreased the AtaT cytotoxicity (Fig. 3a). S76A and S78A also decreased the AtaT toxicity, and the mutation of lysines to alanines in α3 (K77A/K79A/K81A/K82A) reduced the AtaT toxicity to the same extent as Y144F. R95A decreased the AtaT toxicity to a similar level as Y144F, and F30A also affected the AtaT toxicity. Y85A abolished the AtaT toxicity (Fig. 3b). These results suggest that the above-mentioned residues are important for tRNAf^Met^ recognition during the acetylation of Met–tRNAf^Met^. The effects of the mutations on the suppression of growth arrest in liquid medium (Fig. 3a, b) were also confirmed by the suppression of growth inhibition on agar plates (Fig. 3c).

### Acetylation of aminoacyl-tRNAs by AtaT in vivo

**Fig. 4:**
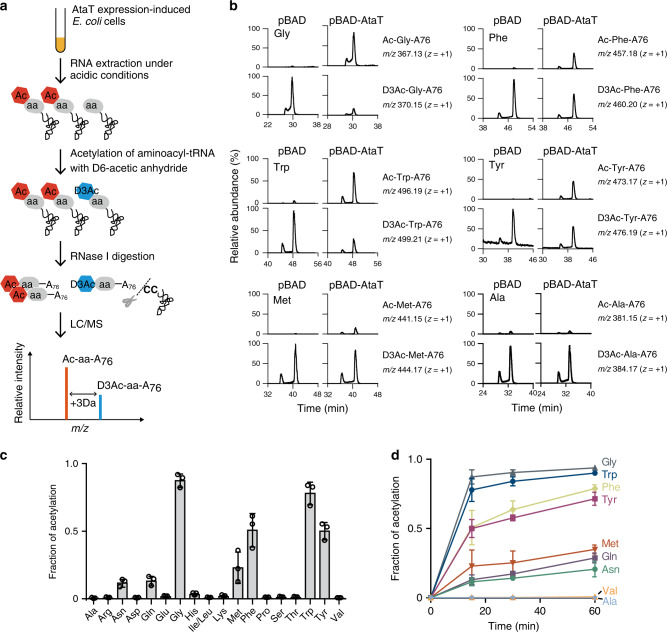
Acetylation of aminoacyl-tRNAs by AtaT in vivo. **a** Schematic diagram of the LC/MS detection and quantification of acetyl-aminoacyl-tRNAs, produced by the action of AtaT in vivo. **b** LC/MS analysis of RNase I-digested fragments of acetyl-aminoacyl-tRNAs after 15 min of induced AtaT expression (pBAD-AtaT) and the negative control (pBAD). Extracted ion chromatograms of the proton adducts corresponding to Ac-Gly-A76 (*m/z* = 367.13), Ac-Trp-A76 (*m/z* = 496.19), Ac-Phe-A76 (*m/z* = 457.18), Ac-Tyr-A76 (*m/z* = 473.17), Ac-Met-A76 (*m/z* = 441.15), and Ac-Ala-A76 (*m/z* = 381.15), derived from Ac-Gly-tRNA^Gly^, Ac-Trp-tRNA^Trp^, Ac-Phe-tRNA^Phe^, Ac-Tyr-tRNA^Tyr^, Ac-Met-tRNA^Met^, and Ac-Ala-tRNA^Ala^ isoacceptors, respectively. The two peaks observed for each Ac-aa-A76 are likely attributable to structural isomers of 3′-acetyl-aminoacyl-A76 and 2′-acetyl-aminoacyl-A76, as 3′-O-methyl and 2′-O-methyl nucleotides are known to be separated in a similar manner^40^. **c** Fractions of aminoacyl-tRNAs acetylated by the action of AtaT in vivo. The amounts of Ac-aa-A76 and D3Ac-aa-A76, after 15 min of AtaT expression induction, were quantified, and the fractions of individual aminoacyl-tRNAs acetylated by AtaT in vivo were estimated as the ratio of Ac-aa-A76 to the sum of the amounts of Ac-aa-A76 and D3Ac-aa-A76. The bars in the graph are SD of three independent (*n* = 3) experiments, and the data are presented as mean values ± SD. **d** Time courses of the fractions of aminoacyl-tRNAs acetylated by AtaT in vivo. The bars in the graphs are SD of three independent experiments.

Although AtaT was initially shown to acetylate the α-amino group of Met–tRNAf^Met^ in vitro (Fig. 1b)^13^, there is no direct evidence for Met–tRNAf^Met^ acetylation by AtaT in vivo. To evaluate the in vivo target of aminoacyl–tRNAs for acetylation by AtaT, AtaT was expressed in *E. coli* and the intracellular aminoacyl–tRNAs were analyzed by LC/MS^26^ (Fig. 4a). *E. coli* cells were harvested after the induction of AtaT expression, using the arabinose-inducible pBAD system^27^. Upon arabinose addition, the growth of AtaT expressing cells was suppressed, as compared to that of *E. coli* cells bearing the empty pBAD plasmid.

To analyze intracellular aminoacyl-tRNAs, the RNA fraction was extracted under acidic and cold conditions, and subsequently acetylated by stable isotopic acetic anhydride-D_6_ [(CD_3_CO)_2_O; D is deuterium] to chemically acetylate the remaining aminoacyl-tRNAs. As a result, all aminoacyl-tRNAs were converted into either intracellularly acetylated aminoacyl-tRNAs (Ac-aa-tRNAs) by AtaT or chemically acetylated aminoacyl-tRNAs (D_3_Ac-aa-tRNAs). The RNAs were then digested with RNase I, and Ac-aa-A76 (A76 is the 3′-terminal adenosine of the tRNA molecule) and D_3_Ac-aa-A76 of each amino acid species were detected by LC/MS. The scheme of the analysis is illustrated in Fig. 4a.

The LC/MS analyses of RNase I-digested RNAs prepared from cells with AtaT induction revealed the molecular masses corresponding to Ac-Gly-A76, Ac-Trp-A76, Ac-Tyr-A76, Ac-Phe-A76, Ac-Gln-A76, Ac-Asn-A76, and Ac-Met-A76, derived from Ac-Gly-tRNA^Gly^, Ac-Trp-tRNA^Trp^, Ac-Tyr-tRNA^Tyr^, Ac-Phe-tRNA^Phe^, Ac-Gln-tRNA^Gln^, Ac-Asn-tRNA^Asn^, and Ac-Met-tRNA^Met^ isoacceptors, respectively. Thus, AtaT acetylates not only Met-tRNA^Met^, but also Gly-tRNA^Gly^, Trp-tRNA^Trp^, Tyr-tRNA^Tyr^, Phe-tRNA^Phe^, Gln-tRNA^Gln^, and Asn-tRNA^Asn^ (Fig. 4b, Supplementary Fig. 4).

Quantification of the ratio of Ac-aa-A76 and D_3_Ac-aa-A76 enables an estimation of the fraction of aminoacyl-tRNAs acetylated by AtaT among cellular aminoacyl-tRNAs (Fig. 4c). The time course of the cellular acetylation by AtaT activity showed the rapid acetylation of Gly-tRNA^Gly^ and Trp-tRNA^Trp^. At 15 min after the induction, about 80% of the Gly-tRNA^Gly^ and Trp-tRNA^Trp^ isoacceptors in cells were already acetylated (Fig. 4d). The fractions of acetylated aminoacyl-tRNAs of Tyr-tRNA^Tyr^ and Phe-tRNA^Phe^ were around 50% at 15 min, and reached 70% at 60 min. For Met-tRNA^Met^, about 30% of Met-tRNA^Met^ was acetylated after 60 min induction. Since elongator Met-tRNAm^Met^ is not a preferred substrate of AtaT in vitro (Fig. 1b), the acetylated Met-tRNA^Met^ would be attributed to Met-tRNAf^Met^. We also detected N-formyl methionyl adenosine (formyl-Met-A76) from formyl-Met-tRNAf^Met^. The levels of formyl-Met-A76 were not significantly altered between AtaT-induced and control *E. coli* cells (Supplementary Fig. 5). The acetylation ratios of Gln-tRNA^Gln^ and Asn-tRNA^Asn^ were low even at 60 min after the induction, indicating that they are probably minor AtaT substrates.

### Acetylation of aminoacyl-tRNAs by AtaT in vitro

**Fig. 5:**
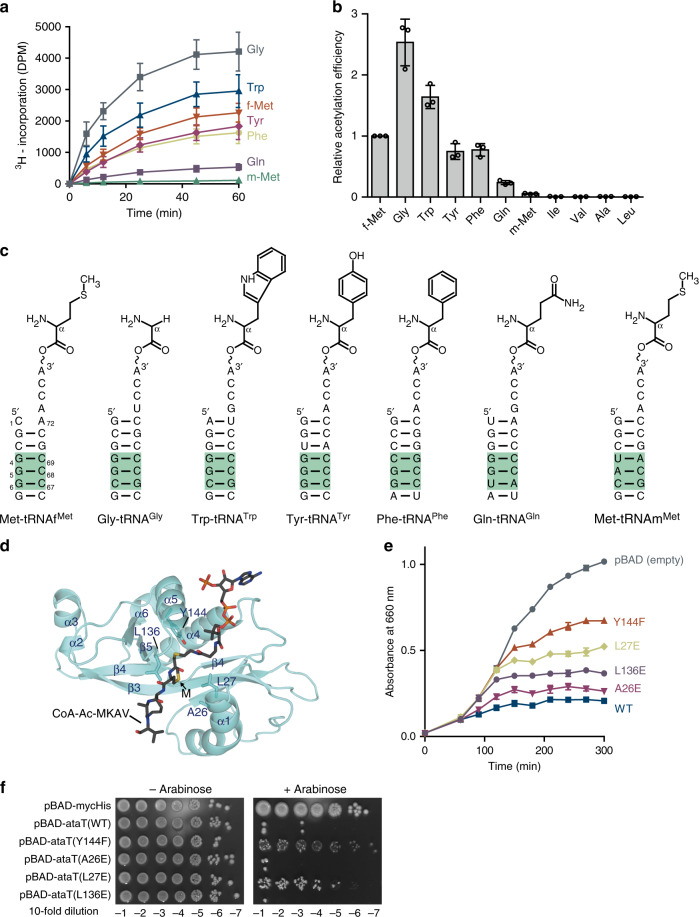
Acetylation of aminoacyl-tRNAs by AtaT in vitro. **a** Time courses of the acetylations of Gly-tRNA^Gly^ (Gly), Trp-tRNA^Trp^ (Trp), Tyr-tRNA^Tyr^ (Tyr), Phe-tRNA^Phe^ (Phe), Met-tRNAf^Met^ (f-Met), Met-tRNAm^Met^ (m-Met), Leu-tRNA^Leu^ (Leu), Ala-tRNA^Ala^ (Ala), Ile-tRNA^Ile^(Ile), and Val-tRNA^Val^(Val) by AtaT in vitro. The respective aminoacyl-tRNAs (10 μM) were used for the acetylation by AtaT. **b** Quantification of the acetylation efficiencies in **a**. The relative initial velocities of the acetylation of various aminoacyl-tRNAs were measured. Acetylation of the Met-tRNAf^Met^ transcript was taken as 1.0. The bars in the graph are SD of three independent (*n* = 3) experiments, and the data are presented as mean values ± SD. **c** Nucleotide sequences of acceptor helices and chemical structures of aminoacyl-moieties of aminoacyl-tRNAs acetylated by AtaT. **d** Model of CoA-Ac-MKAV in the catalytic pocket of the AtaT structure. CoA-Ac-MKAV is shown in a stick model. **e** Growth curves of *E. coli* MG1655 transformed with pBAD-ataT and its variants with mutations in the putative aminoacyl moiety binding site. **f** Overnight cultures of *E. coli* MG1655 transformed with pBAD-ataT and its variants in **e** were serially diluted, and the dilutions were spotted on LB agar plates containing 50 μg mL^−1^ ampicillin supplemented with 0.2% (w/v) arabinose or without arabinose. The plates were incubated at 37 °C overnight.

The LC/MS analysis of the intracellular aminoacyl-tRNAs upon AtaT expression revealed that, in addition to Met-tRNAf^Met^, Gly-tRNA^Gly^, Try-tRNA^Trp^, Tyr-tRNA^Tyr^, and Phe-tRNA^Phe^ are efficiently acetylated in vivo by AtaT (Fig. 4c). To verify the results, the acetylation of those aminoacyl-tRNA species (10 μM) by AtaT was assessed in vitro. The *K*_m_ value of Met-tRNAf^Met^ for acetylation by AtaT was estimated to be ~30 μM (Fig. 1b). Thus, the 10 μM concentration of various aminoacyl-tRNAs for the in vitro assays would be sufficiently sensitive to evaluate the specificity of AtaT for aminoacyl-tRNAs. The results revealed the efficient acetylation of the isoacceptors of Gly-tRNA^Gly^, Trp-tRNA^Trp^, Tyr-tRNA^Tyr^, and Phe-tRNA^Phe^ by AtaT (Fig. 5a, b). The acetylation efficiencies of Gly-tRNA^Gly^ and Trp-tRNA^Trp^ by AtaT were about two-times and three-times higher than that of Met-tRNAf^Met^, respectively. The acetylation efficiencies of the Tyr-tRNA^Tyr^ and Phe-tRNA^Phe^ isoacceptors were almost the same as that of Met-tRNAf^Met^. Gln-tRNA^Gln^ isoacceptors were also acetylated by AtaT, but with a relatively lower efficiency of <25% of Met-tRNAf^Met^. Since tryptophan, tyrosine, and phenylalanine, but not glycine, are aromatic hydrophobic amino acids (Fig. 5c), we tested the acetylation of other aminoacyl-tRNAs charged with hydrophobic amino acid residues, such as Val-tRNA^Val^, Ile-tRNA^Ile^, Ala-tRNA^Ala^, and Leu-tRNA^Leu^ isoacceptors. Neither Val-tRNA^Val^, Ile-tRNA^Ile^, Ala-tRNA^Ala^ nor Leu-tRNA^Leu^ was acetylated by AtaT in vitro, and these aminoacyl-tRNAs were also not acetylated by AtaT in vivo (Figs. 4c and 5b). The in vitro acetylation analyses showed similar substrate preferences of AtaT as those in vivo, and thus AtaT has a broader specificity toward aminoacyl-tRNAs than initially described^13^.

The superimposition of the AtaT structure onto the structure of the protein N-terminal acetyltransferase, NatF, complexed with a bisubstrate analog, CoA-Ac-MKAV^28^, identified the residues that may constitute the pocket for the aminoacyl moiety side chain in AtaT. In the model of AtaT complexed with CoA-Ac-MKAV (Fig. 5d), the methionine (M) side chain of CoA-Ac-MKAV is proximal to A26, L27, and L136. These hydrophobic residues would constitute the pocket for the aminoacyl-moiety of aminoacyl-tRNAs. The L27E mutation suppressed the toxicity of AtaT on the solid and liquid LB liquid media to the same extent as the Y144F catalytic mutation, and the L136E mutation weakly suppressed the toxicity of AtaT in the liquid medium (Fig. 5e, f). Thus, the hydrophobic and aromatic side chains of the aminoacyl moieties of aminoacyl-tRNAs, Trp-tRNA^Trp^, Tyr-tRNA^Tyr^, Phe-tRNA^Phe^, and Met-tRNAf^Met^, would be recognized by these hydrophobic residues in the active site of AtaT. The recognition of Gly-tRNA^Gly^ is discussed below.

Since the three consecutive G–C pairs (G4G5G6:C67C68C69) at the bottom-half of the acceptor helix are important for Met–tRNAf^Met^ acetylation (Fig. 1e, g), we compared the nucleotide sequences of the acceptor helices of the aminoacyl–tRNAs acetylated by AtaT (Fig. 5c). tRNA^Gly^, tRNA^Trp^, tRNA^Tyr^, and tRNA^Phe^ are rich in G–C pairs at the bottom halves of their acceptor helices. tRNA^Tyr^ has three consecutive G–C pairs, while tRNAf^Met^, and tRNA^Gly^, tRNA^Trp^, and tRNA^Phe^ have two consecutive G–C pairs and one C–G pair at this position. tRNA^Gln^ has two consecutive G–C pairs and one A–U pair (Supplementary Fig. 6). Thus, this finding is consistent with the biochemical and structural analyses (Figs. 1c–e and 2a) showing that the nucleotide sequences at the bottom halves of the acceptor helices are important for the acetylation of aminoacyl-tRNAs by AtaT.

## Discussion

In this study, we showed that, in addition to Met-tRNAf^Met^, AtaT can acetylate isoacceptors of Gly-tRNA^Gly^, Trp-tRNA^Trp^, Tyr-tRNA^Tyr^, and Phe-tRNA^Phe^ efficiently, and Gln-tRNA^Gln^ to some extent, in vivo and in vitro (Figs. 4 and 5). In particular, Gly-tRNA^Gly^ and Trp-tRNA^Trp^ are more efficiently acetylated than Met-tRNAf^Met^, and Tyr-tRNA^Tyr^ and Phe-tRNA^Phe^ are acetylated as efficiently as Met-tRNAf^Met^ in vitro. Therefore, the substrate specificity of AtaT toward aminoacyl-tRNAs is broader than initially described. Moreover, after AtaT expression in *E. coli*, the level of formyl-Met-tRNAf^Met^ in the cells did not change (Supplementary Fig. 5). The previous study suggested that the acetylation of the α-amino group of the aminoacyl-moiety of Met-tRNAf^Met^ by AtaT inhibits the formylation of the α-amino group of the aminoacyl-moiety of Met-tRNAf^Met^ by formylase^13^. Thus, AtaT was assumed to block the translation initiation step, because acetylated Met-tRNAf^Met^ cannot interact with translation initiation factor 2. After methionylation of tRNAf^Met^ by methionyl-tRNA synthetase, Met-tRNAf^Met^ is subsequently formylated and used for translation initiation. Under the conditions in which AtaT is expressed in *E. coli*, AtaT and formylase would both compete for the Met-tRNAf^Met^ substrate. The *K*_m_ values of Met-tRNAf^Met^ toward the formylase and AtaT are 0.2 ± 0.1^22^ and 29.6 ± 3.8 μM (Fig. 1b), respectively. Thus, the affinity of Met-tRNAf^Met^ toward the formylase is around two orders of magnitude larger than that of Met-tRNAf^Met^ toward AtaT for acetylation. Therefore, even after the induction of AtaT in *E. coli*, the level of fMet-tRNAf^Met^ would not be drastically decreased and the translation initiation would not be inhibited. Consistent with this notion, as revealed by the LC/MS analyses of aminoacyl-tRNAs isolated from *E. coli* with AtaT induction, AtaT does not change the level of fMet-tRNAf^Met^ in vivo (Supplementary Fig. 5). Thus, AtaT inhibits global translation, rather than the translation initiation step. In the previous report^13^, to identify the target aminoacyl-tRNA of AtaT, various synthetic tRNA transcripts were used as substrates for aminoacylation by the *E. coli* S100 fraction, followed by acetylation by AtaT. Some of the synthetic tRNA transcripts would not have been aminoacylated efficiently, as they lacked modified nucleosides in them, and thus only the acetylation of initiator Met-tRNAf^Met^ was significantly observed.

The structural analysis of AtaT complexed with Ac-Met-tRNAf^Met^ and the biochemical analysis of AtaT using tRNAf^Met^ variant transcripts suggested that the acceptor stem region of tRNA interacts with the dimeric AtaT, and the consecutive three G–C pairs in the bottom half of the acceptor stem are important for the acetylation by AtaT (Figs. 1d g, 2a). Consistent with this finding, the aminoacyl-tRNAs acetylated by AtaT in vivo, other than Met-tRNAf^Met^, also carry two or three G–C pairs in the bottom halves of their acceptor stems (Fig. 5c). At the reaction stage, α3 in AtaT should specifically interact with the G–C pairs in the bottom half of the acceptor stem. The mutation of C1–A72 to C1–G72 in the tRNAf^Met^ transcript did not abolish acetylation by AtaT (Fig. 1d, e). Therefore, the mispair at the top of the acceptor helix is not necessarily required for the acetylation of aminoacyl tRNAs. On the other hand, mutations of C1–A72 to A1–C72 or G1–C72 in the tRNAf^Met^ transcript decreased the acetylation efficiency by AtaT (Fig. 1d, e). A purine base at position 72 might strengthen the base stacking with the base at 73 to facilitate the relocation of the aminoacyl-moiety in the active site of AtaT. Gly-tRNA^Gly^, Tyr-tRNA^Tyr^, and Phe-tRNA^Phe^ have G1–C72 base pairs at the top of their acceptor stems and are efficiently acetylated by AtaT in vivo and in vitro (Figs. 4c, d and 5a, b). The G–C pairs in the bottom half of the acceptor stems of tRNA^Gly^, tRNA^Tyr^, or tRNA^Phe^ might be sufficient to overcome the negative effect of the G1–C72 base pair at the top of the acceptor stem, for acetylation by AtaT. Alternatively, as described below, the interactions of the aminoacyl-moieties of Gly-tRNA^Gly^, Tyr-tRNA^Tyr^, or Phe-tRNA^Phe^ with AtaT are strong enough to overcome the negative effect of the G1–C72 base pair at the top of the acceptor helix, for acetylation by AtaT.

While Met-tRNAf^Met^ is acetylated by AtaT, misaminoacylated tRNAf^Met^s, such as Val-tRNAf^Met^UAC or Ile-tRNAf^Met^GAU, are not acetylated (Fig. 1c), suggesting that the aminoacyl-moieties of aminoacyl-tRNAs are also recognized by AtaT. The structure of the binding pocket for the aminoacyl-moiety of aminoacyl-tRNA in AtaT would be suitable for the accommodation of hydrophobic aromatic residues (Fig. 5d–f). The side chains of tryptophan, tyrosine, and phenylalanine would snugly fit into the hydrophobic pocket of the AtaT catalytic site. The hydrophobic side chain of methionine would also be accommodated in the hydrophobic pocket. While the side chain of the aminoacyl-moiety of Gly-tRNA^Gly^ is a hydrogen atom and thus not hydrophobic, the small hydrogen atom could enter the hydrophobic pocket of AtaT without any restraints. On the other hand, although the valine and isoleucine side chains of mischarged Val-tRNAf^Met^UAC and Ile-tRNAf^Met^GAU, respectively, are hydrophobic, they are small and cannot be snugly accommodated within the hydrophobic pocket, and thus neither Val-tRNAf^Met^ nor Ile-tRNAf^Met^ is acetylated by AtaT (Fig. 1c). Since glutamine is not a hydrophobic residue, the side chain of the aminoacyl-moiety of Gln-tRNA^Gln^ cannot snugly fit in the hydrophobic pocket of AtaT, and tRNA^Gln^ has two G:C pairs and one U–A pair in the bottom half of the acceptor stem (Fig. 5c). Thus Gln-tRNA^Gln^ is not acetylated as efficiently as other substrate aminoacyl-tRNAs by AtaT. The selection mechanism of the aminoacyl-moiety side-chain of an aminoacyl-tRNA is homologous to that of ItaT^26^ and other related aminoacyl-tRNA protein transferases, such as leucyl/phenylalanyl-tRNA-protein transferase^29,30^. The size and shape of the binding pocket of AtaT for the aminoacyl moiety of an aminoacyl-tRNA are suitable for the accommodation of hydrophobic aromatic residues, except Gly-tRNA^Gly^.

Taken together, the selection of an aminoacyl-tRNA for acetylation by AtaT is governed by both the specific sequence in the acceptor stem and the properties of the aminoacyl-moiety side chains of aminoacyl-tRNAs. The mechanism governing the affinity of AtaT for an aminoacyl-tRNA is analogous to that observed in the interaction between an aminoacyl-tRNA and translation elongation factor Tu (EF-Tu). The affinity of EF-Tu for an aminoacyl-tRNA is modulated by the aminoacyl moiety and tRNA sequence, and the sum of the affinities for the aminoacyl-moiety and the RNA body determines the total affinity of EF-Tu for an aminoacyl-tRNA^31–33^. While EF-Tu binds with approximately the same affinity to all elongator aminoacyl-tRNAs, AtaT binds with different affinity to aminoacyl-tRNAs. The sum of the affinities of AtaT for the hydrophobic aminoacyl-moiety and the tRNA acceptor stem sequence would determine the suitability of a substrate to be acetylated by AtaT.

GNAT family toxins were reported to have different specificities for aminoacyl-tRNAs^12–16^. Recently, ItaT from *E. coli* HS was shown to acetylate Ile-tRNA^Ile^, Val-tRNA^Val^, and Met-tRNA^Met^ in vivo and in vitro^26^. Since there is no consensus sequence in the acceptor stem of the substrate isoaccepting tRNAs, ItaT presumably selects its substrate aminoacyl-tRNA by mainly recognizing the aminoacyl moieties of aminoacyl-tRNAs, and the size and shape of the hydrophobic pocket of ItaT are suitable for the accommodation of specific aminoacyl moieties^26^. The hydrophobic pocket of AtaT for the aminoacyl-moieties of aminoacyl-tRNAs would be larger than that of ItaT and suitable for the accommodation of bulkier hydrophobic aromatic residues. A comparison of the electrostatic surface potential of AtaT with those of TacT and ItaT revealed that ItaT has a distinct distribution of positively charged residues (Supplementary Fig. 7), suggesting that the affinity of ItaT for tRNA itself might be weaker than that of AtaT or the mechanism of tRNA recognition might be different from that of AtaT. This also can explain why Gly-tRNA^Gly^ is not acetylated by ItaT, while AtaT acetylates Gly-tRNA^Gly^. Although the distributions of the positively charged regions of AtaT and TacT are similar, the residues of AtaT involving interactions with the bottom half of the acceptor helix of tRNAf^Met^ (S76, K77, S78, and K79) are not conserved beyond the clade of closely related GNAT toxins, such as KacT from *Klebsiella pneumoniae* (Supplementary Fig. 8). Therefore, while the binding mode of TacT with an aminoacyl-tRNA might be similar to that of AtaT, the mechanism of specific tRNA recognition and the properties of the binding pocket for the aminoacyl-moiety would be different. Thus, TacT would have a distinct preference from AtaT for aminoacyl-tRNAs. Further detailed comparative analyses of the in vivo targets and structures of these GNAT toxins complexed with tRNA will clarify the overall view of the molecular mechanisms of these toxins.

## Methods

### Plasmids

The plasmid pET-ataT(G108D) for overexpression of AtaT (G108D), and the plasmid pET-ataRT for overexpression of the AtaRT complex were previously described^21^. The pBAD-ataT plasmid, for evaluation of the toxic activity of AtaT in vivo, was described previously^21^. Mutations in pBAD-ataT were introduced by the overlap PCR method. The DNA fragment encoding glutaminyl-tRNA synthetase, glnS, was PCR amplified from the *E. coli* genome and cloned into the NdeI and XhoI sites of pET-22b. The plasmids for expression of methionyl-tRNA synthetase and isoleucyl-tRNA synthetase were described previously^26^. The plasmids for the overexpression of other aminoacyl-tRNA synthetases (ARSs: AlaRS, ValRS, LeuRS, GlyRS, TrpRS, TyrRS, and PheRS) were kind gifts from Dr. Shimizu (RIKEN, Japan). The oligonucleotide sequences used for the plasmid constructions or mutageneses are listed in Supplementary Table S1.

### Protein expression and purification

The AtaT (G108D) protein was overexpressed in *E. coli* BL21(DE3) cells (Novagen-Merck Millipore) and purified as described^21^. The wild-type AtaT was expressed with AtaR in *E. coli* BL21(DE3) cells and purified from the AtaT–AtaR complex as described^21^.

### In vitro transcription of mutant tRNAf^Met^

A precursor tRNA bearing a 5′ leader was synthesized by T7 RNA polymerase and processed by RNaseP, comprising the M1 RNA and C5 protein. The synthetic *rnp*B gene encoding M1 RNA under the T7 promoter was synthesized and purchased from Eurofins, Japan, and M1 RNA was synthesized by T7 RNA polymerase. The synthetic *rnp*A gene encoding the C5 protein was also synthesized and purchased from Eurofins, Japan and cloned into the NdeI and XhoI sites of pET-22b. The C5 protein was overexpressed in *E. coli* BL21(DE3) and purified essentially as described. The pre-tRNA transcript was processed in a mixture containing 100 nM M1 RNA, 100 nM C5 protein, 50 mM Tris–HCl, pH 7.4, 10 mM MgCl_2_, and 100 mM NH_4_Cl at 37 °C for 3 h, followed by phenol-extraction and isopropanol precipitation. The processed mature tRNAs were dissolved in 20 mM Tris–HCl, pH 7.4, buffer containing 200 mM NaCl, applied to a Resource Q column (GE Healthcare, Japan), and separated by a linear NaCl gradient (0.2–1.0 M) in the buffer.

The DNA fragments encoding template sequences for in vitro transcription, and the sequences of the synthetic *rnp*A and *rnp*B genes are listed in Supplementary Table 2.

### Purification of overexpressed tRNAs in *E. coli* cells

tRNAf^Met^, tRNAm^Met^, tRNA^Gly^, tRNA^Trp^, tRNA^Phe^, tRNA^Tyr^, tRNA^Gln^, tRNA^Ala^, tRNA^Ile^, and tRNA^Val^ were overexpressed in *E. coli* transformed with the pBSTNAV3 plasmids encoding the respective tRNA genes, and purified as described^22,34^. The synthetic DNA fragments encoding the *E. coli* tRNA^Gly^, tRNA^Trp^, tRNA^Tyr^, and tRNA^Gln^ genes were inserted between the SacI and PstI sites of the pBSTNAV3 plasmid. DNA fragment sequences are listed in Supplementary Table 2. The pBSTNAV3 plasmids encoding tRNAf^Met^, tRNAm^Met^, tRNAf^Met^GCU, tRNAf^Met^UAC, tRNA^Leu^, tRNA^Ile^, tRNA^Val^, and tRNA^Ala^ were described previously^22,23,26^. The *E. coli* JM101Tr strain was transformed with the pBSTNAV3 plasmid and cultured in 2 × YT medium containing 50 μg mL^−1^ ampicillin, at 37 °C for 24 h. The total tRNA fraction was prepared as described, with modifications^22,34^. After deacylation of the aminoacyl-tRNAs, total RNAs were dissolved in buffer containing 20 mM Tris–HCl, pH 7.4, 0.1 mM EDTA, and 8 mM Mg_2_(OAc), loaded on a HiLoad 16/10 Q-Sepharose HP column (GE Healthcare, Japan), and separated by a linear NaCl gradient (0.2–1.0 M) in the buffer. Fractions enriched with the overexpressed tRNA were detected by aminoacylation, using the respective cognate aminoacyl-tRNA synthetase and radiolabeled amino acid, pooled and isopropanol-precipitated. The tRNA fractions prepared as described above were aminoacylated by their cognate aminoacyl-tRNA synthetases, and the amounts of the enriched isoacceptor tRNAs in the respective tRNA fractions were measured.

### In vitro acetylation assay

tRNA was first aminoacylated in a mixture containing 20 mM Tris–HCl, pH 7.4, 150 mM KCl, 7 mM MgCl_2_, 10 mM β-mercaptoethanol, 15 μM tRNA, 200 μM methionine, and 2 μM MetRS, at 37 °C for 60 min. The acetylation reaction was started by adding a mixture containing 160 μM [acetyl-^3^H] Coenzyme A (10 Ci mmol^−1^, Perkin Elmer, Japan) and 20 nM AtaT. An aliquot of the reaction was spotted onto a Whatman 3MM filter (GE Healthcare, Japan) and the radioactivities on the filters were measured with a liquid scintillation counter (Hitachi Aloka Medical).

For the kinetic analysis, methionyl-tRNAf^Met^ or tRNAm^Met^ was extracted by phenol saturated with 300 mM NaOAc, pH 5.2, and applied to a NAP-5 column (GE Healthcare, Japan) for removal of the remaining ATP, and then the eluate was precipitated by ethanol. Met-tRNAs were dissolved in 5 mM NaOAc, pH 5.2, quantified by measuring the *A*_260_ values, and diluted to a series of concentrations. Met-tRNAf^Met^ and Met-tRNAm^Met^ were incubated with 20 and 250 nM AtaT, respectively, in a mixture containing 10 mM Tris–HCl, pH 7.0, 100 mM KCl, 5 mM MgCl_2_, 10 mM β-mercaptoethanol, and 160 μM [acetyl-^3^H] Coenzyme A (10 Ci mol^−1^, Perkin Elmer, Japan), and the initial reaction velocities were measured. For analysis of aminoacyl-tRNA specificity of AtaT in vitro, 10 μM of aminoacyl-tRNA were used for the assay.

### Crystallization and structural determination of AtaT complexed with acetyl-methionyl-tRNAf^Met^

The purified tRNAf^Met^ was charged with methionine by MetRS, in a reaction mixture containing 20 mM Tris–HCl, pH 7.4, 150 mM KCl, 7 mM MgCl_2_, 10 mM β-mercaptoethanol, 20 mL^−1^ *A*_260_ tRNAf^Met^, 1 mM methionine, 2 mM ATP, and 1 μM MetRS, at 37 °C for 1 h. The reaction product was extracted by cold phenol saturated with 300 mM NaOAc, pH 5.2, and applied to a NAP-5 column (GE Healthcare) at 4 °C for removal of the remaining ATP, and the eluate was precipitated by ethanol. Methionyl tRNAf^Met^ was dissolved in 600 μL of 0.3 M NaOAc, chemically acetylated by adding 30 μL of acetic anhydride, and incubated for 15 min on ice, and this procedure was repeated four times. The reaction mixture was then ethanol precipitated and loaded onto a Nucleosil 300-5 C8 column (Macherey-Nagel) to separate the acetyl-methionyl-tRNAf^Met^ from residual methionyl- and deacyl-tRNAf^Met^. Acetyl-methionyl-tRNAfMet was dissolved in buffer containing 400 mM NaCl, 10 mM MgOAc, and 20 mM NH_4_OAc (pH 5.0), and eluted with a linear methanol gradient (0–36%) in the buffer.

A mixture of 40 μM AtaT (G108D) protein and 40 μM acetyl-methionyl-tRNAf^Met^ was incubated on ice for 30 min, in buffer containing 10 mM Tris–HCl, pH 7.0, 100 mM NaCl, and 5 mM β-mercaptoethanol. The solution was mixed with a reservoir solution containing 100 mM Hepes, pH 6.8, 0.2 mM sodium formate, 18% (v/v) PEG3350, and 0.5% (v/v) jeffamine, and crystals were generated by the hanging drop vapor diffusion method at 20 °C. Crystals were dehydrated by an incubation against the reservoir with an increased concentration of PEG3350 to 33% (v/v), before the diffraction experiment. Data sets were collected at beamline 17A at the Photon Factory at KEK, Japan. The crystals were flash-cooled in 1.1× concentrated reservoir solution, supplemented with 25% (v/v) ethylene glycol as a cryoprotectant. The two datasets were collected from the crystals harvested from a single drop, and they were indexed, integrated, scaled with XDS, and merged using XSCALE^35^. The initial phase was determined by the molecular replacement method, using the Phaser program. As the search model, the structures of the AtaT dimer (PDB ID: 6GTP)^20^ and fMet-tRNAf^Met^ (PDB ID: 2FMT)^24^ were used. The structures were refined with phenix.refine^36^, and manually modified with Coot^37^. Representative images of the electron density are shown in Supplementary Fig. 2.

### In vivo toxicity assay for AtaT and its variants

*E. coli* strain MG1655, purchased from NBRP: *E. coli* (NIG, Japan; ME7986), was transformed with pBAD-AtaT or its variants, inoculated in LB containing 50 μg mL^−1^ ampicillin in the presence of 1% (w/v) glucose, and cultured at 37 °C. To examine the toxicities of AtaT and its variants in the liquid medium, the overnight cultures were diluted to an OD_660_ of 0.02 in fresh liquid LB containing 50 μg mL^−1^ ampicillin and supplemented with 1% (w/v) glucose or 0.2% (w/v) arabinose. The cultures were continued at 37 °C, and the OD_660_ values of the cultures were measured. To test the toxicities of AtaT by the spot assay, the overnight LB cultures were serially diluted, and 3 μL portions of the diluents were spotted on LB agar plates containing 50 μg mL^−1^ ampicillin supplemented with 0.2% (w/v) arabinose, and then the plates were incubated overnight at 37 °C.

### Preparation of acetylated aminoacyl-tRNAs from *E. coli* upon AtaT induction

*E. coli* strain MG1655 was transformed with pBAD-AtaT or empty pBAD/Myc-His A (Invitrogen) and cultured overnight. The overnight cultures were diluted to an OD_660_ of 0.02 in fresh liquid LB (4 mL) containing 50 μg/ml ampicillin, and cultured at 37 °C until the *A*_660_ reached 0.2. Then, 0.02% (w/v) arabinose was added. After a 15, 30, or 60 min incubation, the cells were harvested and suspended in buffer containing 50 mM NaOAc, pH 5.0, 0.5 mM EDTA, and 0.2 M NaCl. RNA was extracted by phenol saturated with 300 mM NaOAc, pH 5.2, followed by isopropyl alcohol precipitation. The RNA was dissolved in 250 μL of 200 mM NaOAc, pH 5.0, acetylated by adding acetic anhydride-D_6_ (Sigma Aldrich, Japan)^38^, and then ethanol precipitated and rinsed with 70% cold ethanol. The RNA was dissolved in cold buffer containing 50 mM NaOAc, pH 5.0, 0.5 mM EDTA, and 0.2 M NaCl, and loaded onto a 100 μL Q-Sepharose F.F. (GE Healthcare, Japan) column. The resin was washed with buffer containing 50 mM NaOAc, pH 5.0, 0.5 mM EDTA, and 0.2 M NaCl. tRNA was eluted with buffer containing 50 mM NaOAc, pH 5.0, 0.5 mM EDTA, and 0.6 M NaCl, ethanol precipitated, and rinsed with 70% (v/v) ethanol.

### LC/MS spectrometry

The purified acetylated aminoacyl-tRNAs were digested with RNase One Ribonuclease (Promega, Japan), in a reaction mixture (25 μL volume) containing 25 mM NH_4_OAc and 2.5 units enzyme, at 37 °C for 60 min. The digests were subjected to an LC/MS analysis using a Q Exactive Hybrid Quadrupole-Orbitrap Mass Spectrometer (Thermo Fisher Scientific) equipped with a Dionex UltiMate 3000 LC System (Thermo Fisher Scientific) and an InertSustain C18 column (5 μm, 2.1 × 250 mm, GL Sciences). Elution was carried out using a multi-linear gradient with 5 mM ammonium acetate (pH 5.3) (solvent A) and 60% acetonitrile (solvent B). The following gradient was used: 1–35% B from 0 to 35 min; 35–99% B from 35 to 40 min; 99% B from 40 to 50 min; 99–1% B from 50 to 50.1 min; and 1% B from 50.1 to 60 min. Ions were scanned by the use of the positive polarity mode over an *m/z* range of 110–950^39^.

### Reporting summary

Further information on research design is available in the Nature Research Reporting Summary linked to this article.

## Supplementary information

Supplementary Information

Description of Additional Supplementary Files

Supplementary Data 1

Reporting Summary

## Data Availability

Coordinates and structure factors of AtaT–Ac–Met–tRNAf^Met^ complex have been deposited in the Protein Data Bank, under the accession code 7CHD. As the search model, the structures of the AtaT dimer (PDB ID: 6GTP) and fMet-tRNAf^Met^ (PDB ID: 2FMT) were used. All data are available from the corresponding author upon reasonable request. Source data are provided with this paper.

## Source data

Source Data
