# Supplementary material for: Mechanism of aminoacyl-tRNA acetylation by an aminoacyl-tRNA acetyltransferase AtaT from enterohemorrhagic E. coli
Source: Nat Commun. 2020 Oct 28;11:5438. doi: 10.1038/s41467-020-19281-z (PMC7595197; doi:10.1038/s41467-020-19281-z)
Supplement: Supplementary file 1 — Supplementary Information [file 41467_2020_19281_MOESM1_ESM.pdf]

Supplementary Information

Mechanism of aminoacyl-tRNA acetylation by an aminoacyl-tRNA acetyltransferase AtaT from enterohemorrhagic *E. coli*

Yuka Yashiro, Yuriko Sakaguchi, Tsutomu Suzuki, Kozo Tomita

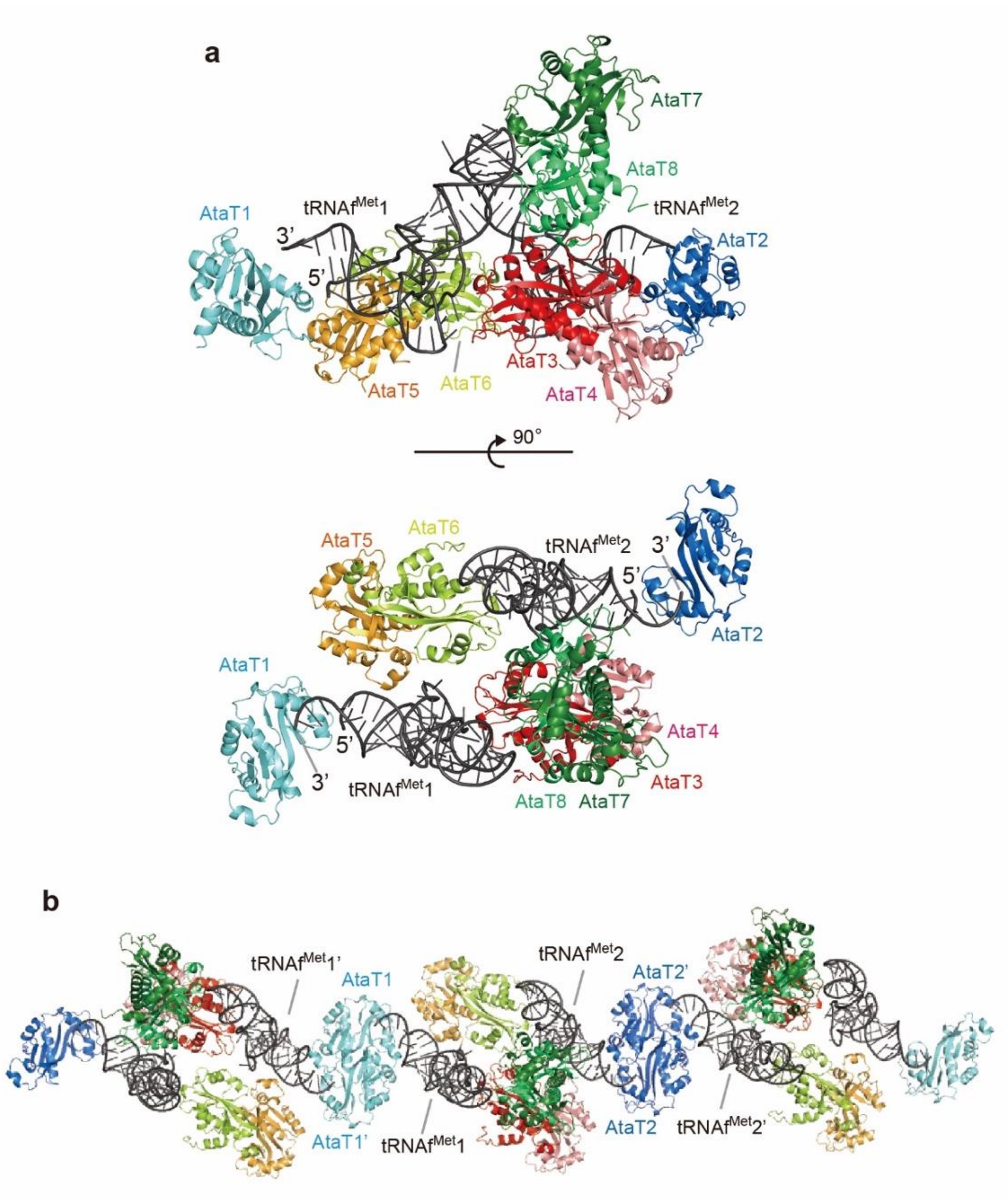

Supplementary Fig. 1: Structure of the AtaT-acMet-tRNAf^{Met} complex in the asymmetric unit cell. (**a**) The structure of the AtaT-acMet-tRNAf^{Met} complex in the asymmetric unit (ASU) of the crystal is shown in a ribbon representation. The ASU contains eight AtaT (AtaT1 - 8) molecules and two acMet-tRNAf^{Met} molecules (acMet-tRNAf^{Met}1 and acMet-tRNAf^{Met}2). The 3' single-stranded regions of tRNA^{fMet}1 and tRNA^{fMet}2 interact with the regions proximal to the catalytic sites of AtaT1 and AtaT2, respectively. The final models of AtaT1, AtaT2, AtaT3, AtaT4, AtaT5, AtaT6, AtaT7 and AtaT8 contain residues 3-172, 3-182, 2-180, 3-172, 5-175, 5-176, 5-172 and 5-172, respectively. (**b**) Ribbon representation of AtaT-acMet-tRNAf^{Met} in the three neighboring ASUs of the crystal. AtaT1 and AtaT2 form dimers with AtaT1' and AtaT2', respectively, through the crystallographic two-fold rotation axis.

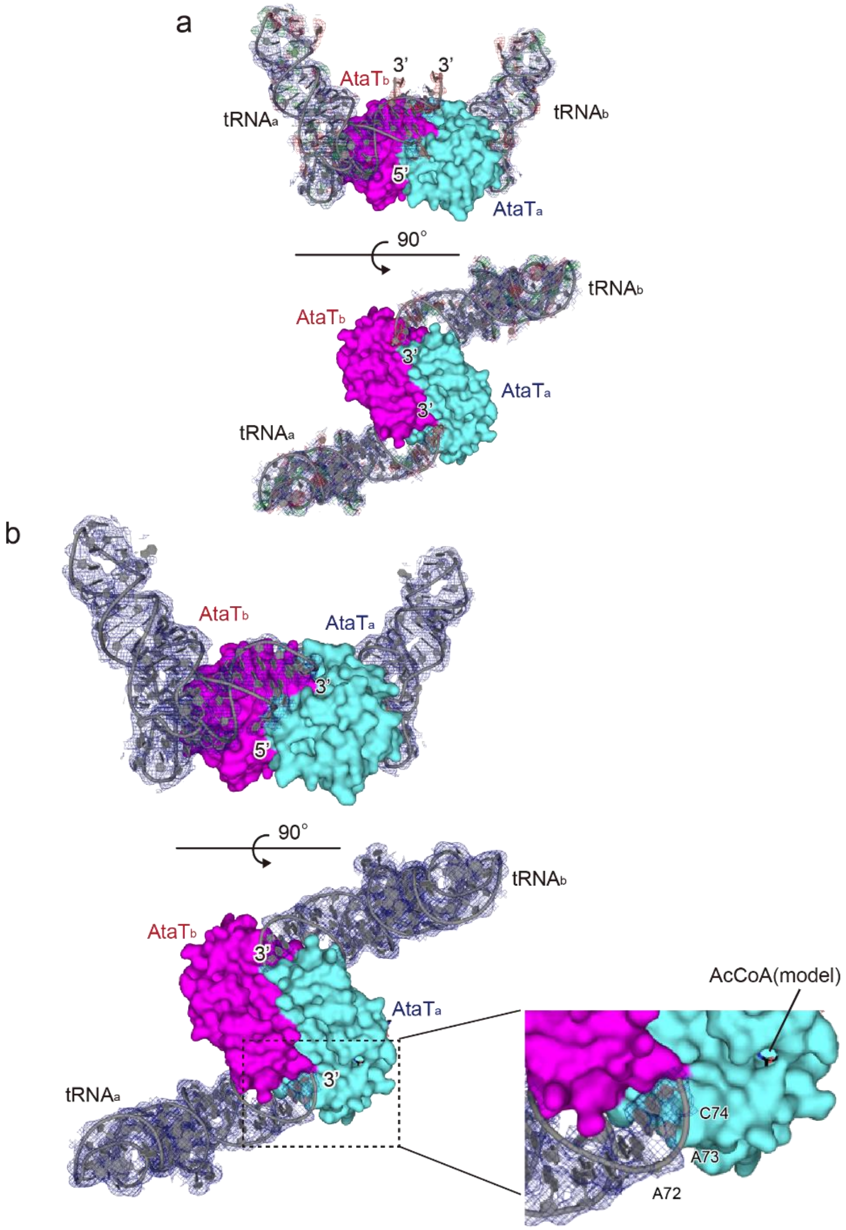

Supplementary Fig. 2: **Electron density maps of acMet-tRNAf^{Met} in the AtaT-acMet-tRNAf^{Met} complex** (**a**) Electron density maps of acMet-tRNAf^{Met} in the AtaT-acMet-tRNAf^{Met} complex after the molecular replacement calculation, using fMet-tRNAf^{Met} (PDB ID: 2FMT) as the search model. The 2*F*o-*F*c electron density map (blue mesh) contoured at 1.0 σ, and the *F*o-*F*c maps contoured at 1.0 and -1.0 σ (green and red meshes, respectively) of acMet-tRNAf^{Met} (tRNAa and tRNAb) in the AtaT-acMet-tRNAf^{Met} complex are shown. (**b**) Composite simulated annealing omit map of acMet-tRNAf^{Met} (tRNAa and tRNAb) in the AtaT-acMet-tRNAf^{Met} complex, contoured at 1.0 σ. Ac-CoA molecules are modeled in the active site of AtaT and shown as sticks. The electron densities corresponding to 3'-C75A76-acMet were not visible in the present structure.

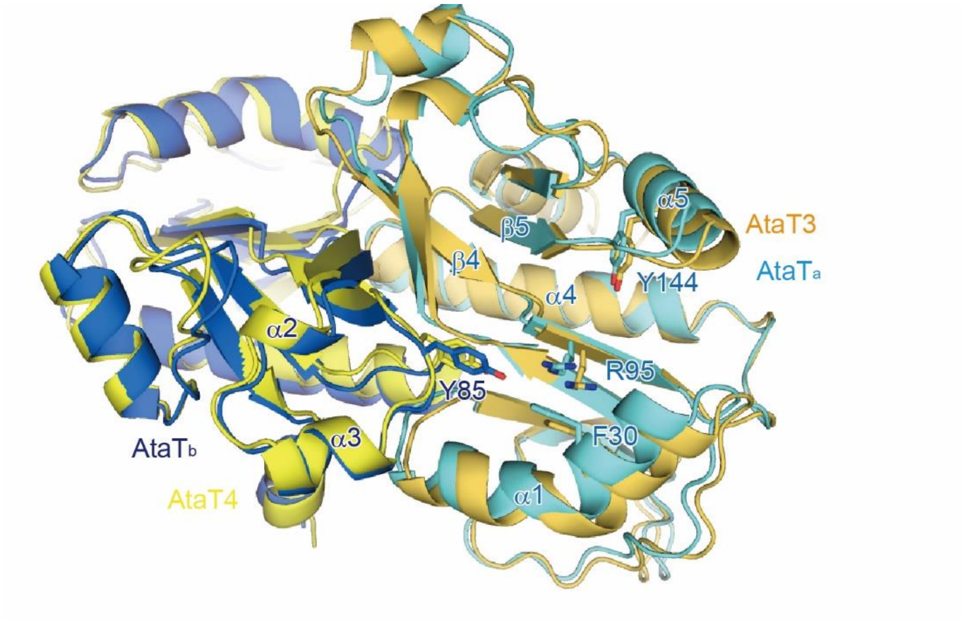

Supplementary Fig. 3: Comparison of the structures of AtaTa-AtaTb in the AtaT- acMet-tRNAf^{Met} complex with AtaT3-AtaT4 in the asymmetric unit cell. Superimposition of the AtaTa-AtaTb structure in the (AtaT)$_2$(ac-Met-tRNAf^{Met})$_2$ complex and AtaT3-AtaT4 in the asymmetric unit cell in Supplementary Fig. 1. AtaTa, AtaTb, AtaT3, and AtaT4 are colored cyan, blue, orange, and yellow, respectively.

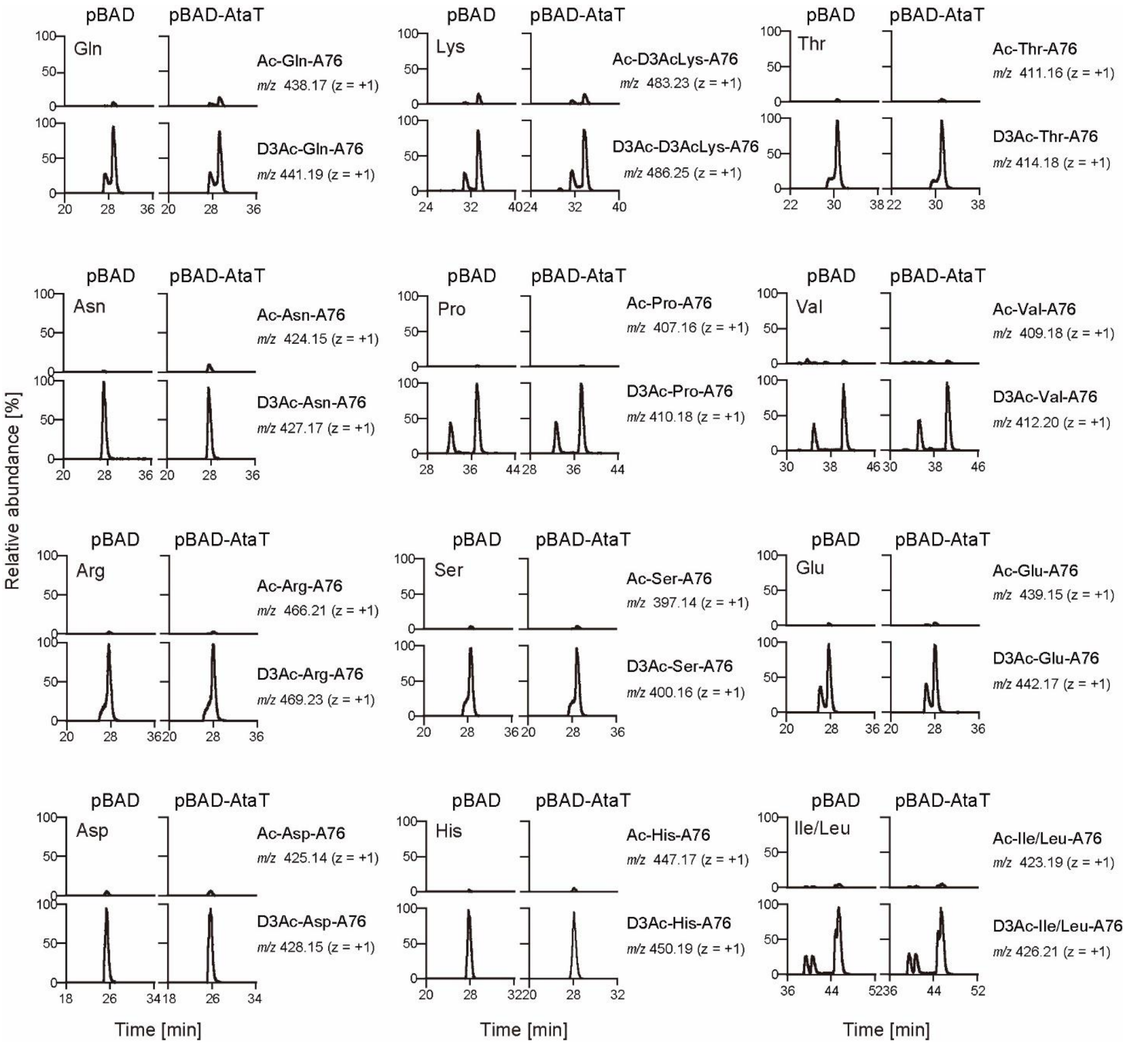

Supplementary Fig. 4: LC/MS analysis of RNase I-digested fragments of acetyl-aminoacyl-tRNAs. LC/MS analysis of RNase I-digested fragments of acetyl-aminoacyl-tRNAs after 15 min of AtaT expression induction. Extracted ion chromatograms (XIC) of the proton adducts corresponding to Ac-aminoacyl-A76 and D_3-aminoacyl-A76 are shown. The subtle peaks corresponding to Ac-aminoacyl-A76 detected in RNA samples from *E. coli* harboring the empty pBAD vector originated from the small fraction of non-deuterated acetic anhydride in the stable isotopic acetic anhydride-D_6. According to the manufacturer's information, the isotopic purity of the stable isotopic acetic anhydride-D_6 was 99.7 %. The molecular mass corresponding to Ac-Cys-A76, derived from Cys-tRNA^{Cys}, was not detected, probably due to the high reactivity of the thiol group, which leads to disulfide bond formation or oxidation by air oxygen during preparation. Cys-tRNA^{Cys} was not efficiently acetylated by AtaT *in vitro.*

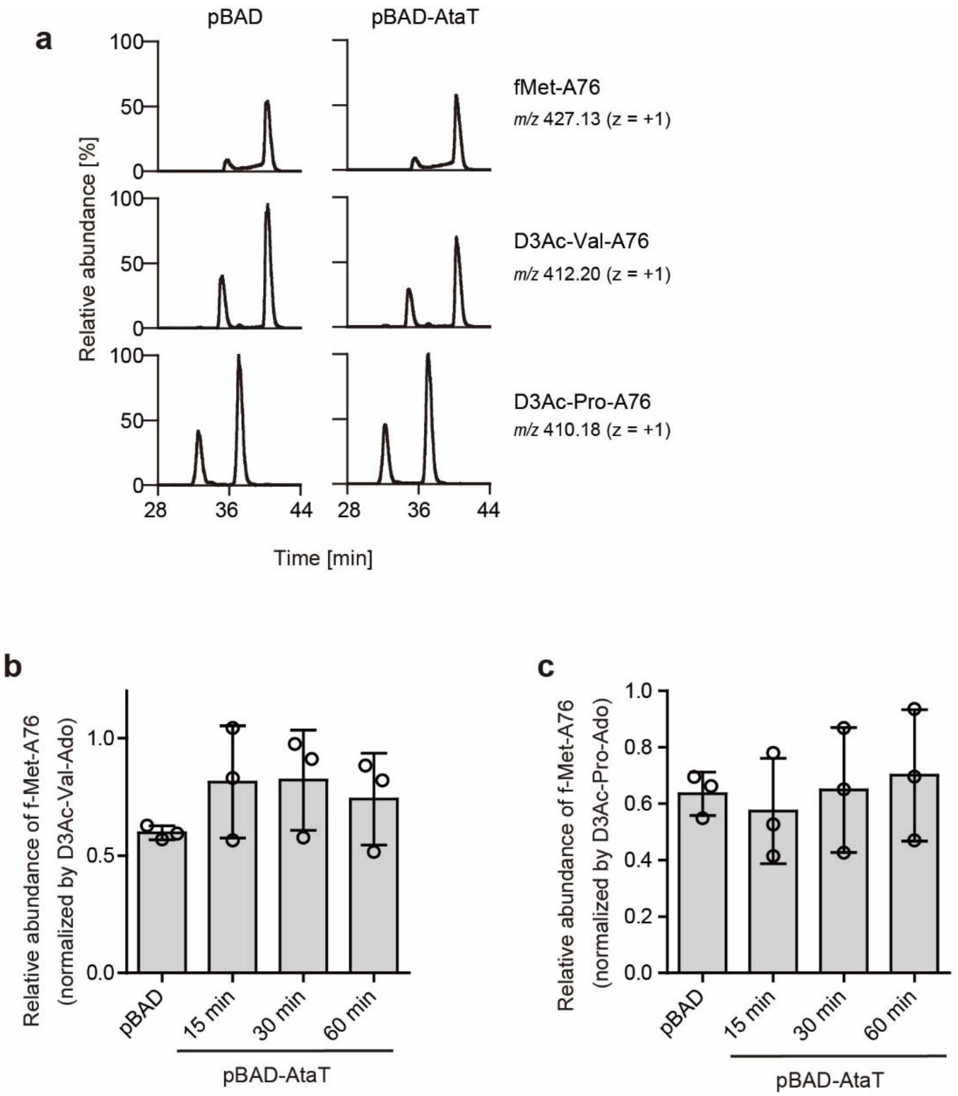

Supplementary Fig. 5: Identification of *N*-formyl-Met-tRNAf^{Met} by LC/MS analysis. (**a**) LC/MS analysis of RNase I-digested fragments of acetyl-aminoacyl-tRNAs after 15 min of AtaT expression induction. Extracted ion chromatograms (XIC) of the proton adducts corresponding to f-Met-A76 (*N*-formyl methionyl-adenosine, *m/z* = 427.13) in RNA preparations from *E. coli* with or without AtaT induction. The intensity of f-Met-A76, D_3Ac-Val-A76, or D_3Ac-Pro-A76 (control) in each sample is expressed relative to the intensity of D_3Ac-Pro-A76. (**b**), (**c**) Relative quantification of fMet-A76. The intensity of f-Met-A76 in RNA preparations from *E. coli*, with or without AtaT induction for the indicated times, was normalized by the intensity of D_3Ac-Val-A76 (**b**) or D_3Ac-Pro-A76 (**c**). The bars in the graphs are SD of three independent experiments. The amounts of fMet-tRNAf^{Met} in the cells are not significantly altered by the AtaT expression. The bars in the graph are SD of three independent (n=3) experiments, and the data are presented as mean values ± SD.

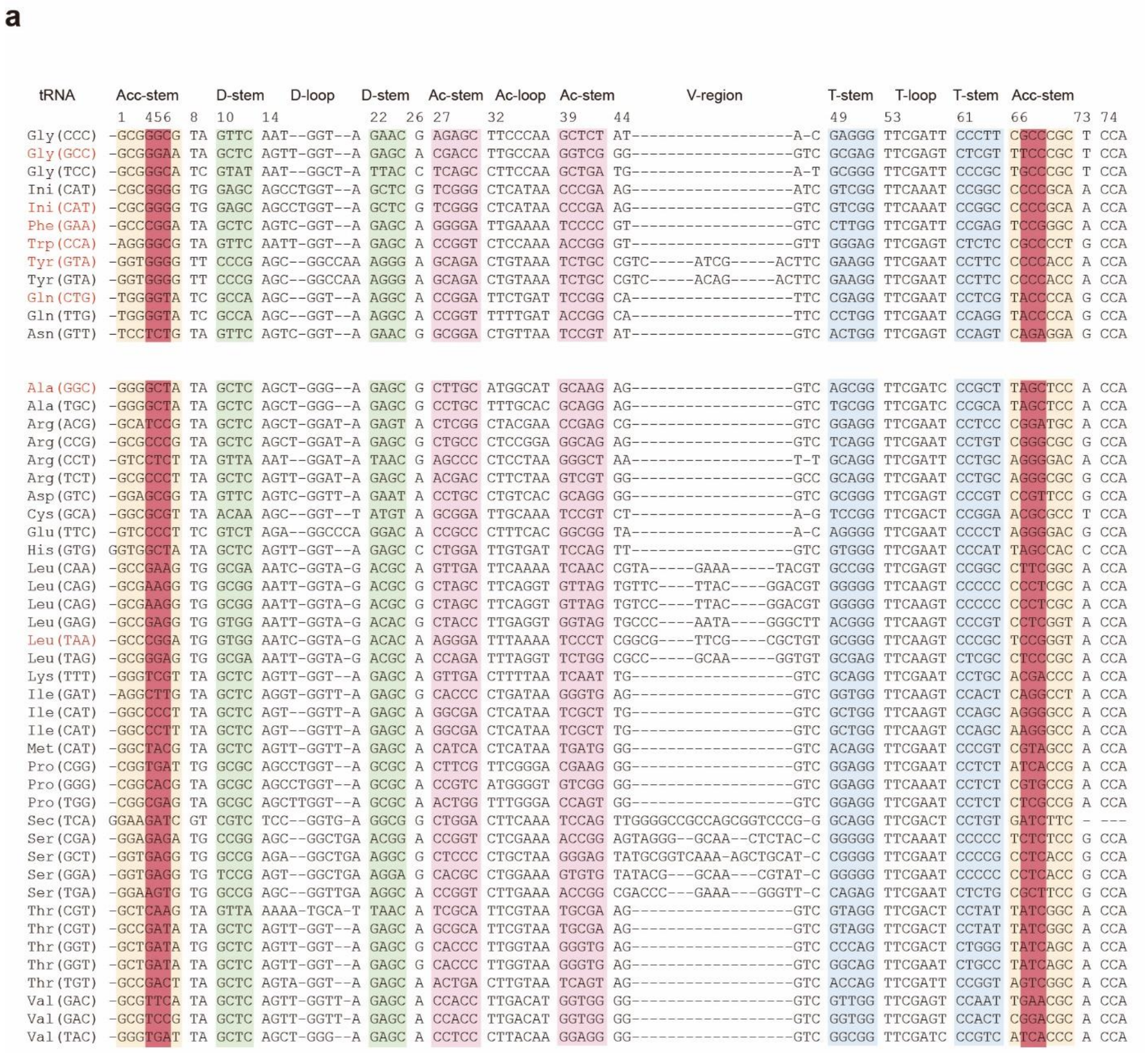

Supplementary Fig. 6: Sequence alignment of *E. coli* tRNA genes. (**a**) *E. coli* K12 and (**b**) *E. coli* O157: H7 tRNA genes are shown. tRNA isoacceptors used for the *in vitro* acetylation assay in this study are colored red.

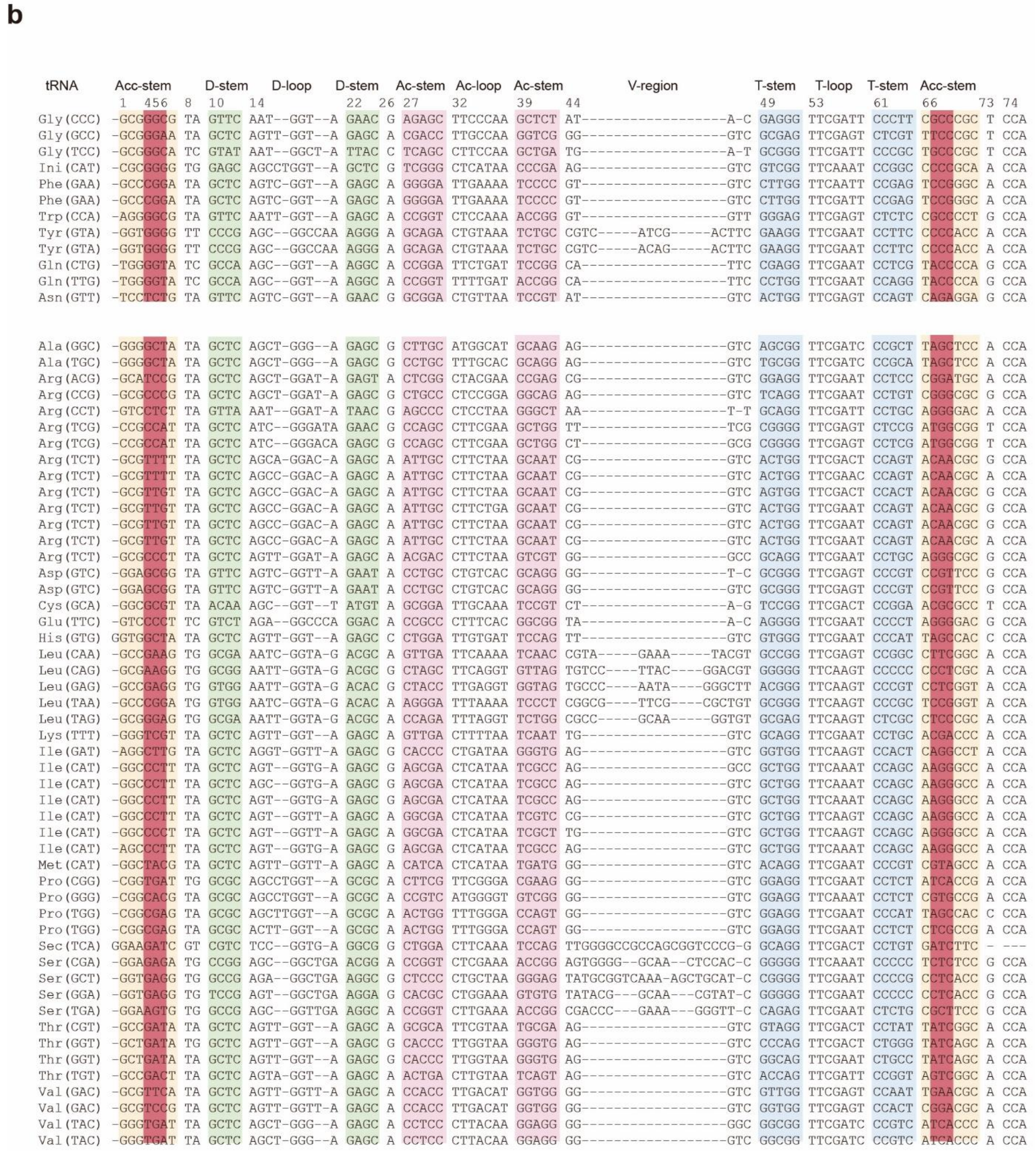

Supplementary Fig. 6: continued

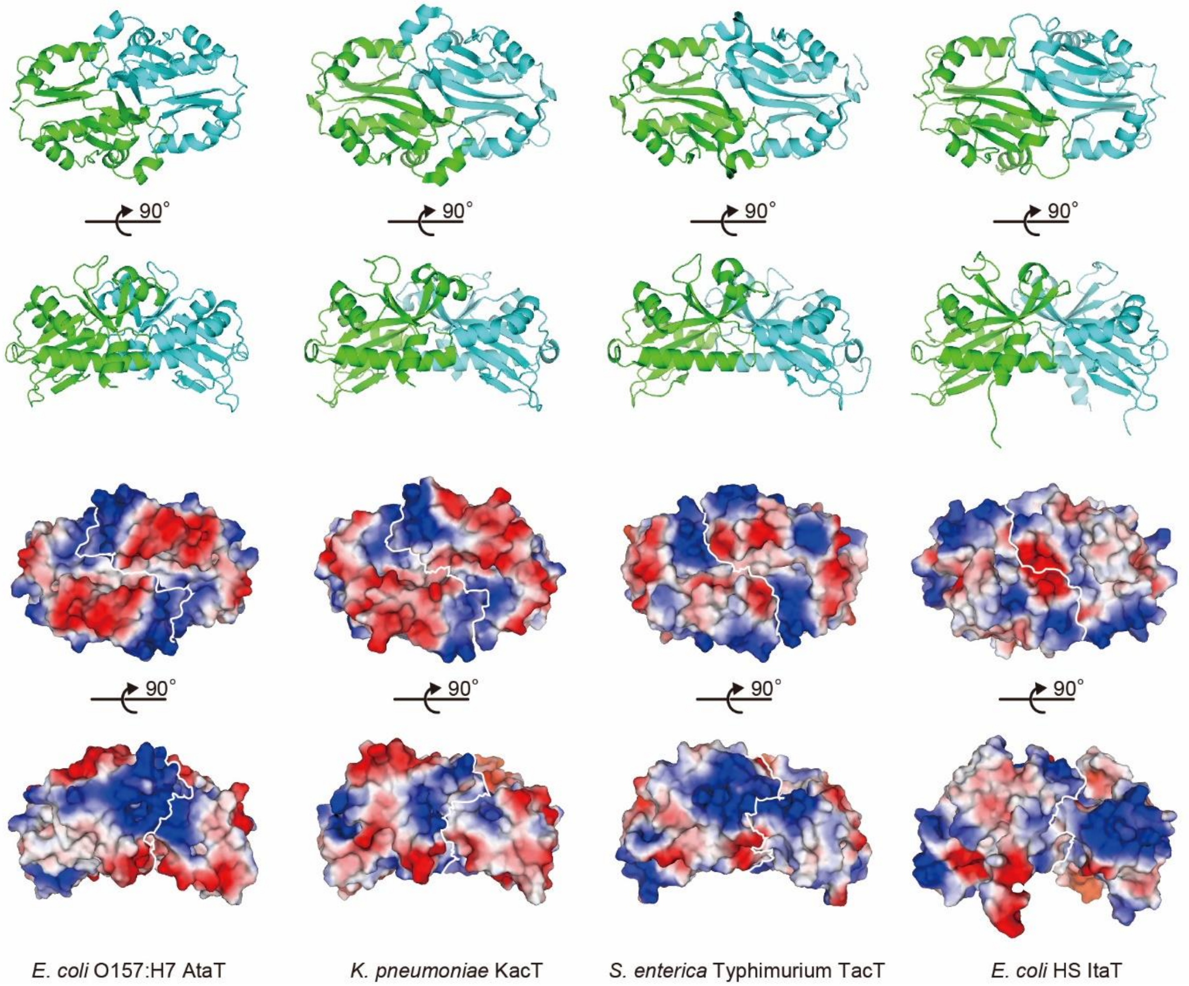

Supplementary Fig. 7: Comparison of the structures of GNAT toxins. Ribbon representations and surface electrostatic potentials of *E. coli* O157: H7 AtaT in the AtaT-acMet-tRNAf^{Met} complex (this study), *K. pneumoniae* KacT (PDB ID: 5XUN) [1], *S. enterica* Typhimurium TacT (PDB ID: 5FVJ) [2], and *E. coli* HS ItaT (PDB ID: 7BYY) [3] are shown. Positively and negatively charged areas are colored blue and red, respectively.

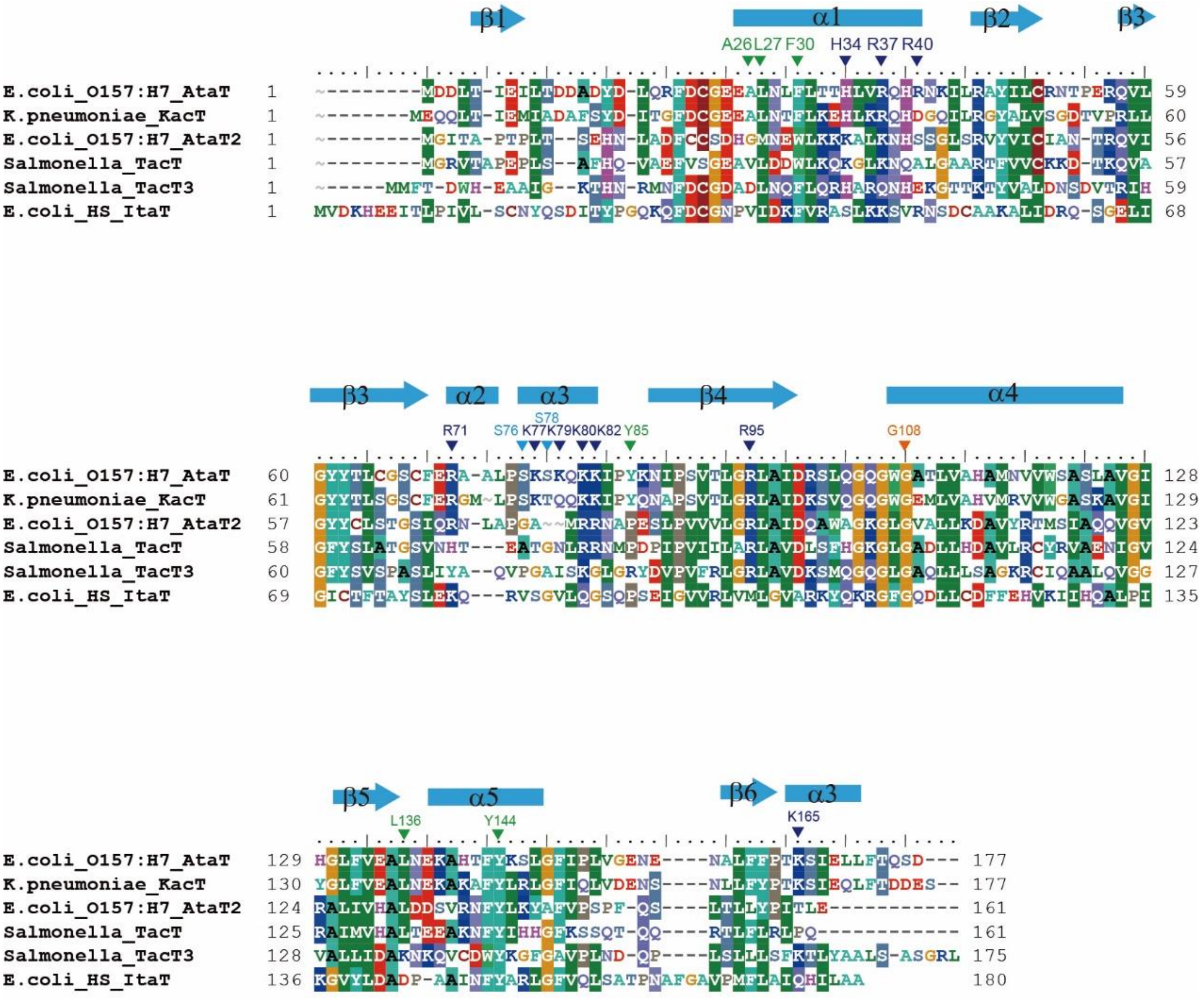

Supplementary Fig. 8: Sequence alignment of GNAT toxins targeting aminoacyl-tRNAs. Amino acid sequences of *E. coli* O157: H7 AtaT [4] and AtaT2 [5], *S. enterica* Typhimurium TacT [2] and TacT3 [6], *K. pneumoniae* KacT [7], and *E. coli* HS ItaT [8] are aligned. The secondary structure elements (α1 - α5 and β1 - β6) of AtaT are depicted above the alignment.

Supplementary Table 1: Synthetic DNA primers used in this study

ataT_F30A_F	GCG CTG ACG ACA CAT CTC GTT CGT C
ataT_F30_R	GAGATTTAACGCTTCCTCGCC
ataT_H34R37R40A_F	CAA CAT GCG AAC AAA ATT CTG CGA GCG TAT ATC
ataT_H34R37R40A_R	CGCAACGAGCGCTGTCGTCAGAAAGAGATTTAACG
ataT_K77K79K81K82A_F	CAG GCG GCG ATT CCC TAC AAA AAT ATT CCC AGC
ataT_K77K79K81K82A_R	CGCCGACGCCGAGGGCAATGCGGCTCG
ataT_Y85A_F	GCG AAA AAT ATT CCC AGC GTT ACT CTT GG
ataT_Y85_R	GGGAATTTTTTCTGTTTCGATTTCGAGG
ataT_R95A_F	GCG CTG GCA ATT GAT CGT TCA TTA CAG
ataT_R95_R	CCCAAGAGTAACGCTGGGAATATTTTTG
ataT_K165A_F	GCG TCC ATT GAA CTG CTT TTT ACA CAG AGC
ataT_K164_R	GGTTGGGAAAAATAACGCATTTTCG
ataT_S76A_F	GCG AAA TCG AAA CAG AAA AAA ATT CCC TAC
ataT_S78A_F	TCG AAA GCG AAA CAG AAA AAA ATT CCC TAC
ataT_S76,78F_R	GGGCAATGCGGCTCGTTCAAAACAAC
ataT_R71A_F	GCG GCC GCA TTG CCC TCG AAA TCG AAA CAG
ataT_R71_R	TTCAAAACAACTGCCGCATAATGTGTAATATCCC
ataT_Y144F_F	TTT AAA TCG CTG GGC TTT ATC CCT TTA GTC GG
ataT_Y144_R	AAACGTATGGGCTTTTTCATTCAGCGCCTCGAC
AtaT_A26E_F	gaa TTA AAT CTC TTT CTG ACG ACA CAT CTC
AtaT_A26_R	TTCCTCGCCGCAGTCGAATCGCTG
AtaT_L27E_F	gaa AAT CTC TTT CTG ACG ACA CAT CTC GTT CGT C
AtaT_L27_R	CGCTTCCTCGCCGCAGTCGAATCG
AtaT_L136E_F	gaa AAT GAA AAA GCC CAT ACG TTT TAT AAA TCG C
AtaT_L136_R	CGCCTCGACAAAAAGACCGTGAATACC
GlnRS_Fw	agctagctcatATGAGTGAGGCAGAAGCCCGCCC
GlnRS_Rv	agctctcgagCTCGCCTACTTTCGCCCAGGTATC

Supplementary Table 2: Synthetic DNA sequences used in this study

***rnpA* gene:**<br>CATATGGTTAAGCTCGCATTTCCCAGGGAGTTACGCTTGTTAACTCCCAGTCAATTCACAT<br>TCGTCTTCCAGCAGCCACAACGGGCTGGCACGCCGCAAATTACCATTCTCGGCCGCCTGA<br>ATTCGCTGGGGCATCCCCGTATCGGTCTTACAGTCGCCAAGAAAAACGTTCGACGCGCCC<br>ATGAACGCAATCGGATTAAACGTCTGACGCGTGAAAGCTTCCGTCTGCGCCAACATGAAC<br>TCCCGGCTATGGATTTCGTGGTGGTGGCGAAAAAGGGGTTGCCGACCTCGATAACCGTG<br>CTCTCTCGGAAGCGTTGGAAAAATTATGGCGCCGCCACTGTCGCCTGGCTCGCGGGTCCC<br>TCGAG
***rnp*B gene (M1 RNA) template:**<br>GAATTCTAATACGACTCACTATAGGGAAGCTGACCAGACAGTCGCCGCTTCGTCGTCGTC<br>CTCTTCGGGGGAGACGGGCGGAGGGGAGGAAAGTCCGGGCTCCATAGGGCAGGGTGCC<br>AGGTAACGCCTGGGGGGGAAACCCACGACCAGTGCAACAGAGAGCAAACCGCCGATGG<br>CCCGCGCAAGCGGGATCAGGTAAGGGTGAAAGGGTGCGGTAAGAGCGCACCGCGCGGC<br>TGGTAACAGTCCGTGGCACGGTAAACTCCACCCGGAGCAAGGCCAAATAGGGGTTCATA<br>AGGTACGGCCCGTACTGAACCCGGGTAGGCTGCTTGAGCCAGTGAGCGATTGCTGGCCT<br>AGATGAATGACTGTCCACGACAGAACCCGGCTTATCGGTCAGTTTCACCTGGCTAGCTAC<br>ATCCAAGCTT
**tRNAf^{Met} WT template:**<br>GAATTCTAATACGACTCACTATAGGGAGACCACAACGGTTTCCCTCTAGACGCGGGGTGG<br>AGCAGCCTGGTAGCTCGTCGGGCTCATAACCCGAAGGTCGTCGGTTCAAATCCGGCCCCC<br>GCAACCAGGCTAGCTACATCCAAGCTT
**tRNAm^{Met} WT template:**<br>GAATTCTAATACGACTCACTATAGGGAGACCACAACGGTTTCCCTCTAGAGGCTACGTAG<br>CTCAGTTGGTTAGAGCACATCACTCATAATGATGGGGTCACAGGTTCGAATCCCGTCGTA<br>GCCACCAGGCTAGCTACATCCAAGCTT
**tRNAf^{Met} A1:C72 template:**<br>GAATTCTAATACGACTCACTATAGGGAGACCACAACGGTTTCCCTCTAGAAGCGGGGTGG<br>AGCAGCCTGGTAGCTCGTCGGGCTCATAACCCGAAGGTCGTCGGTTCAAATCCGGCCCCC<br>GCCACCAGGCTAGCTACATCCAAGCTT
**tRNAf^{Met} C1:G72 template:**<br>GAATTCTAATACGACTCACTATAGGGAGACCACAACGGTTTCCCTCTAGACGCGGGGTGG<br>AGCAGCCTGGTAGCTCGTCGGGCTCATAACCCGAAGGTCGTCGGTTCAAATCCGGCCCCC<br>GCGACCAGGCTAGCTACATCCAAGCTT
**tRNAf^{Met} G1:C72 template:**<br>GAATTCTAATACGACTCACTATAGGGAGACCACAACGGTTTCCCTCTAGAGGCGGGGTGG<br>AGCAGCCTGGTAGCTCGTCGGGCTCATAACCCGAAGGTCGTCGGTTCAAATCCGGCCCCC<br>GCCACCAGGCTAGCTACATCCAAGCTT
**tRNAf^{Met} U4A5C6:G67U68A69 template:**<br>gaattcTAATACGACTCACTATAGGGAGACCACAACGGTTTCCCTCTAGACGCTACGTGGAG<br>CAGCCtGGTAGCTCGTCGGGCTCATAACCCGAAGGtCGTCGGTTCAAATCCGGCCGTAGCA<br>ACCAggctagctaCATCCAAGCTT

Supplementary Table 2: Synthetic DNA sequences used in this study, continued

**tRNAf^{Met} C4C5C6:G67G68G69 template:**<br>gaattcTAATACGACTCACTATAGGGAGACCACAACGGTTTCCCTCTAGACGCCCCGTGGAG<br>CAGCCtGGTAGCTCGTCGGGCTCATAACCCGAAGGtCGTCGGTTCAAATCCGGCCGGGGC<br>AACCAggctagctaCATCCAAGCTT
**tRNAm^{Met} fMet acceptor stem template:**<br>GAATTCTAATACGACTCACTATAGGGAGACCACAACGGTTTCCCTCTAGACGCGGGGTAG<br>CTCAGTTGGTTAGAGCACATCACTCATAATGATGGGGTCACAGGTTCGAATCCCGTCCCC<br>GCAACCAGGCTAGCTACATCCAAGCTT
**tRNAf^{Met} (C4-G69) template**<br>GAATTCTAATACGACTCACTATAGGGAGACCACAACGGTTTCCCTCTAGACGCCGGGTGG<br>AGCAGCCTGGTAGCTCGTCGGGCTCATAACCCGAAGGTCGTCGGTTCAAATCCGGCCCC<br>GGCAACCAGGCTAGCTACATCCAAGCTT
**tRNAf^{Met} (C5-G68) template**<br>GAATTCTAATACGACTCACTATAGGGAGACCACAACGGTTTCCCTCTAGACGCGCGGTGG<br>AGCAGCCTGGTAGCTCGTCGGGCTCATAACCCGAAGGTCGTCGGTTCAAATCCGGCCCG<br>CGCAACCAGGCTAGCTACATCCAAGCTT
**tRNAf^{Met} (C6-G67) template**<br>GAATTCTAATACGACTCACTATAGGGAGACCACAACGGTTTCCCTCTAGACGCGGCGTGG<br>AGCAGCCTGGTAGCTCGTCGGGCTCATAACCCGAAGGTCGTCGGTTCAAATCCGGCCGC<br>CGCAACCAGGCTAGCTACATCCAAGCTT
**tRNA^{Gly} 2 (GCC) gene:**<br>GCGGGAATAGCTCAGTTGGTAGAGCACGACCTTGCCAAGGTCGGGGTCGCGAGTTCGAG<br>TCTCGTTTCCCGCTCCA
**tRNA^{Trp} gene:**<br>AGGGGCGTAGTTCAATTGGTAGAGCACCGGTCTCCAAAACCGGGTGTTGGGAGTTCGAG<br>TCTCTCCGCCCCTGCCA
**tRNA^{Tyr} gene:**<br>GGTGGGGTTCCCGAGCGGCCAAAGGGAGCAGACTGTAAATCTGCCGTCATCGACTTCGA<br>AGGTTCGAATCCTTCCCCCACCACCA
**tRNA^{Gln} 2 gene:**<br>TGGGGTATCGCCAAGCGGTAAGGCACCGGATTCTGATTCCGGCATTCCGAGGTTCGAATC<br>CTCGTACCCCAGCCA

References

1 Qian, H. *et al.* Toxin-antitoxin operon kacAT of Klebsiella pneumoniae is regulated by conditional cooperativity via a W-shaped KacA-KacT complex. *Nucleic acids research* **47**, 7690-7702, doi:10.1093/nar/gkz563 (2019).

2 Cheverton, A. M. *et al.* A Salmonella Toxin Promotes Persister Formation through Acetylation of tRNA. *Molecular Cell* **63**, 86-96, doi:10.1016/j.molcel.2016.05.002 (2016).

3 Zhang, C., Yashiro, Y., Sakaguchi, Y., Suzuki, T. & Tomita, K. Substrate specificities of Escherichia coli ItaT that acetylates aminoacyl-tRNAs. *Nucleic acids research*, doi:10.1093/nar/gkaa487 (2020).

4 Jurenas, D. *et al.* AtaT blocks translation initiation by N-acetylation of the initiator tRNA(fMet). *Nature Chemical Biology* **13**, 640-646, doi:10.1038/nchembio.2346 (2017).

5 Jurenas, D., Garcia-Pino, A. & Van Melderen, L. Novel toxins from type II toxin-antitoxin systems with acetyltransferase activity. *Plasmid* **93**, 30-35, doi:10.1016/j.plasmid.2017.08.005 (2017).

6 Rycroft, J. A. *et al.* Activity of acetyltransferase toxins involved in Salmonella persister formation during macrophage infection. *Nature Communications* **9**, 11, doi:10.1038/s41467-018-04472-6 (2018).

7 Qian, H. L. *et al.* Identification and characterization of acetyltransferase-type toxin-antitoxin locus in Klebsiella pneumoniae. *Molecular Microbiology* **108**, 336-349, doi:10.1111/mmi.13934 (2018).

8 Wilcox, B. *et al.* Escherichia coli ItaT is a type II toxin that inhibits translation by acetylating isoleucyl-tRNAIle. *Nucleic acids research* **46**, 7873-7885, doi:10.1093/nar/gky560 (2018).
