# Supplementary material for: Mechanism of aminoacyl-tRNA acetylation by an aminoacyl-tRNA acetyltransferase AtaT from enterohemorrhagic E. coli
Source: Nat Commun. 2020 Oct 28;11:5438. doi: 10.1038/s41467-020-19281-z (PMC7595197; doi:10.1038/s41467-020-19281-z)
Supplement: Supplementary file 2 — Description of Additional Supplementary Files [file 41467_2020_19281_MOESM2_ESM.pdf]

Description of Additional Supplementary Files

File Name: Supplementary Data 1
Description: XSCALE Log
