# Supplementary material for: Mechanism of aminoacyl-tRNA acetylation by an aminoacyl-tRNA acetyltransferase AtaT from enterohemorrhagic E. coli
Source: Nat Commun. 2020 Oct 28;11:5438. doi: 10.1038/s41467-020-19281-z (PMC7595197; doi:10.1038/s41467-020-19281-z)
Supplement: Supplementary file 3 — Supplementary Data 1 [file 41467_2020_19281_MOESM3_ESM.pdf]

```
***** XSCALE ***** (VERSION Mar 15, 2019  BUILT=20191015)  27-Dec-2019
Author: Wolfgang Kabsch
Copy licensed until 30-Sep-2020 to
 academic users for non-commercial applications
No redistribution.


******************************************************************************
                              CONTROL CARDS
******************************************************************************

OUTPUT_FILE=fae-ip.ahkl
 INPUT_FILE= ./XDS_ASCII_osc1.HKL
 INPUT_FILE= ./XDS_ASCII_osc2.HKL

THE DATA COLLECTION STATISTICS REPORTED BELOW ASSUMES:
SPACE_GROUP_NUMBER=   18
UNIT_CELL_CONSTANTS=   269.83    68.28   136.18  90.000  90.000  90.000

*****  4 EQUIVALENT POSITIONS IN SPACE GROUP # 18 *****

   If x',y',z' is an equivalent position to x,y,z, then
       x'=x*ML(1)+y*ML( 2)+z*ML( 3)+ML( 4)/12.0
       y'=x*ML(5)+y*ML( 6)+z*ML( 7)+ML( 8)/12.0
       z'=x*ML(9)+y*ML(10)+z*ML(11)+ML(12)/12.0

   #   1  2  3  4    5  6  7  8    9 10 11 12
   1   1  0  0  0    0  1  0  0    0  0  1  0
   2  -1  0  0  0    0 -1  0  0    0  0  1  0
   3   1  0  0  6    0 -1  0  6    0  0 -1  0
   4  -1  0  0  6    0  1  0  6    0  0 -1  0


ALL DATA SETS WILL BE SCALED TO ./XDS_ASCII_osc1.HKL


******************************************************************************
                    READING INPUT REFLECTION DATA FILES
******************************************************************************

DATA    MEAN       REFLECTIONS        INPUT FILE NAME
```

SET# INTENSITY ACCEPTED REJECTED

1	0.1512E+02	333497	0	./XDS_ASCII_osc1.HKL
2	0.1118E+02	334739	0	./XDS_ASCII_osc2.HKL

****

OVERALL SCALING AND CRYSTAL DISORDER CORRECTION

****

CORRELATIONS BETWEEN INPUT DATA SETS AFTER CORRECTIONS

DATA SETS #i	#j	NUMBER OF COMMON REFLECTIONS	CORRELATION BETWEEN i,j	RATIO OF COMMON INTENSITIES (i/j)	B-FACTOR BETWEEN i,j
1	2	25440	0.995	1.4825	-4.2490

K*EXP(B*SS) = Factor applied to intensities
SS = (2sin(theta)/lambda)^2

K	B	DATA SET NAME
1.000E+00	0.000	./XDS_ASCII_osc1.HKL
1.483E+00	-4.250	./XDS_ASCII_osc2.HKL

****

CORRECTION FACTORS AS FUNCTION OF IMAGE NUMBER & RESOLUTION

****

RECIPROCAL CORRECTION FACTORS FOR INPUT DATA SETS MERGED TO
OUTPUT FILE: fae-ip.ahkl

THE CALCULATIONS ASSUME FRIEDEL'S_LAW= TRUE
TOTAL NUMBER OF CORRECTION FACTORS DEFINED 2760
DEGREES OF FREEDOM OF CHI^2 FIT 448413.0
CHI^2-VALUE OF FIT OF CORRECTION FACTORS 0.666
NUMBER OF CYCLES CARRIED OUT 3

CORRECTION FACTORS for visual inspection by XDS-Viewer DECAY_001.cbf
XMIN= 0.2 XMAX= 359.8 NXBIN= 69
YMIN= 0.00043 YMAX= 0.06907 NYBIN= 20
NUMBER OF REFLECTIONS USED FOR DETERMINING CORRECTION FACTORS

237890

CORRECTION FACTORS for visual inspection by XDS-Viewer DECAY_002.cbf
XMIN= 0.2 XMAX= 359.8 NXBIN= 69
YMIN= 0.00043 YMAX= 0.06910 NYBIN= 20
NUMBER OF REFLECTIONS USED FOR DETERMINING CORRECTION FACTORS
236083

****
CORRECTION FACTORS AS FUNCTION OF X (fast) & Y(slow) IN THE DETECTOR PLANE
****

RECIPROCAL CORRECTION FACTORS FOR INPUT DATA SETS MERGED TO
OUTPUT FILE: fae-ip.ahkl

THE CALCULATIONS ASSUME FRIEDEL'S_LAW= TRUE
TOTAL NUMBER OF CORRECTION FACTORS DEFINED 9522
DEGREES OF FREEDOM OF CHI^2 FIT 448200.2
CHI^2-VALUE OF FIT OF CORRECTION FACTORS 0.663
NUMBER OF CYCLES CARRIED OUT 3

CORRECTION FACTORS for visual inspection by XDS-Viewer MODPIX_001.cbf
XMIN= 391.3 XMAX= 2017.0 NXBIN= 69
YMIN= 425.9 YMAX= 2054.9 NYBIN= 69
NUMBER OF REFLECTIONS USED FOR DETERMINING CORRECTION FACTORS
237890

CORRECTION FACTORS for visual inspection by XDS-Viewer MODPIX_002.cbf
XMIN= 392.2 XMAX= 2019.4 NXBIN= 69
YMIN= 426.0 YMAX= 2054.7 NYBIN= 69
NUMBER OF REFLECTIONS USED FOR DETERMINING CORRECTION FACTORS
236083

****
CORRECTION FACTORS AS FUNCTION OF IMAGE NUMBER & DETECTOR SURFACE POSITION
****

RECIPROCAL CORRECTION FACTORS FOR INPUT DATA SETS MERGED TO
OUTPUT FILE: fae-ip.ahkl

THE CALCULATIONS ASSUME FRIEDEL'S_LAW= TRUE
TOTAL NUMBER OF CORRECTION FACTORS DEFINED 1794
DEGREES OF FREEDOM OF CHI^2 FIT 448413.0
CHI^2-VALUE OF FIT OF CORRECTION FACTORS 0.661
NUMBER OF CYCLES CARRIED OUT 3

CORRECTION FACTORS for visual inspection by XDS-Viewer ABSORP_001.cbf
XMIN= 0.2 XMAX= 359.8 NXBIN= 69
DETECTOR_SURFACE_POSITION= 1204 1240
DETECTOR_SURFACE_POSITION= 1480 1517
DETECTOR_SURFACE_POSITION= 928 1517
DETECTOR_SURFACE_POSITION= 928 964
DETECTOR_SURFACE_POSITION= 1480 964
DETECTOR_SURFACE_POSITION= 1829 1500
DETECTOR_SURFACE_POSITION= 1463 1867
DETECTOR_SURFACE_POSITION= 945 1867
DETECTOR_SURFACE_POSITION= 579 1500
DETECTOR_SURFACE_POSITION= 579 981
DETECTOR_SURFACE_POSITION= 945 614
DETECTOR_SURFACE_POSITION= 1463 614
DETECTOR_SURFACE_POSITION= 1829 981
NUMBER OF REFLECTIONS USED FOR DETERMINING CORRECTION FACTORS
237890

CORRECTION FACTORS for visual inspection by XDS-Viewer ABSORP_002.cbf
XMIN= 0.2 XMAX= 359.8 NXBIN= 69
DETECTOR_SURFACE_POSITION= 1206 1240
DETECTOR_SURFACE_POSITION= 1482 1517
DETECTOR_SURFACE_POSITION= 929 1517
DETECTOR_SURFACE_POSITION= 929 964
DETECTOR_SURFACE_POSITION= 1482 964
DETECTOR_SURFACE_POSITION= 1831 1500
DETECTOR_SURFACE_POSITION= 1465 1866
DETECTOR_SURFACE_POSITION= 947 1866
DETECTOR_SURFACE_POSITION= 580 1500
DETECTOR_SURFACE_POSITION= 580 981
DETECTOR_SURFACE_POSITION= 947 614
DETECTOR_SURFACE_POSITION= 1465 614
DETECTOR_SURFACE_POSITION= 1831 981
NUMBER OF REFLECTIONS USED FOR DETERMINING CORRECTION FACTORS
236083

****
CORRECTION PARAMETERS FOR THE STANDARD ERROR OF REFLECTION INTENSITIES
****

The variance v0(I) of the intensity I obtained from counting statistics is replaced by v(I)=a*(v0(I)+b*I^2). The model parameters a, b are chosen to minimize the discrepancies between v(I) and the variance estimated from sample statistics of symmetry related reflections. This model implicates an asymptotic limit ISa=1/SQRT(a*b) for the highest I/Sigma(I) that the experimental setup can produce (Diederichs (2010) Acta Cryst D66, 733-740). Often the value of ISa is reduced from the initial value ISa0 due to systematic errors showing up by comparison with other data sets in the scaling procedure. (ISa=ISa0=-1 if v0 is unknown for a data set.)

a	b	ISa	ISa0	INPUT DATA SET
7.198E-01	7.589E-03	13.53	16.48	./XDS_ASCII_osc1.HKL
7.363E-01	9.231E-03	12.13	14.88	./XDS_ASCII_osc2.HKL

FACTOR TO PLACE ALL DATA SETS TO AN APPROXIMATE ABSOLUTE SCALE 0.347050E+05
(ASSUMING A PROTEIN WITH 50% SOLVENT)

****
STATISTICS OF SCALED OUTPUT DATA SET : fae-ip.ahkl
FILE TYPE: XDS_ASCII MERGE=FALSE FRIEDEL'S_LAW=TRUE

76 OUT OF 668236 REFLECTIONS REJECTED
668160 REFLECTIONS ON OUTPUT FILE

****
DEFINITIONS:
R-FACTOR
observed = (SUM(ABS(I(h,i)-I(h))))/(SUM(I(h,i)))
expected = expected R-FACTOR derived from Sigma(I)

COMPARED = number of reflections used for calculating R-FACTOR

I/SIGMA = mean of intensity/Sigma(I) of unique reflections
(after merging symmetry-related observations)
Sigma(I) = standard deviation of reflection intensity I
estimated from sample statistics

R-meas = redundancy independent R-factor (intensities)
Diederichs & Karplus (1997), Nature Struct. Biol. 4, 269-275.

CC(1/2) = percentage of correlation between intensities from
random half-datasets. Correlation significant at
the 0.1% level is marked by an asterisk.
Karplus & Diederichs (2012), Science 336, 1030-33
Anomal Corr = percentage of correlation between random half-sets
of anomalous intensity differences. Correlation
significant at the 0.1% level is marked.
SigAno = mean anomalous difference in units of its estimated
standard deviation (|F(+)-F(-)|/Sigma). F(+), F(-)
are structure factor estimates obtained from the
merged intensity observations in each parity class.
Nano = Number of unique reflections used to calculate
Anomal_Corr & SigAno. At least two observations
for each (+ and -) parity are required.

SUBSET OF INTENSITY DATA WITH SIGNAL/NOISE >= -3.0 AS FUNCTION OF RESOLUTION

RESOLUTION LIMIT	NUMBER OF REFLECTIONS OBSERVED	UNIQUE	POSSIBLE	COMPLETENESS OF DATA	R-FACTOR observed	R-FACTOR expected	COMPARED	I/SIGMA	R-meas	CC(1/2)	Anomal Corr	SigAno	Nano
17.01	5896	323	342	94.4%	8.3%	8.5%	5895	31.78	8.6%	100.0*	-26	0.676	177
12.03	12394	585	585	100.0%	5.8%	6.9%	12394	37.69	6.0%	100.0*	-10	0.685	408
9.82	17085	711	711	100.0%	7.1%	7.8%	17085	35.93	7.2%	99.9*	-12	0.712	546
8.51	21145	825	825	100.0%	9.7%	10.0%	21145	28.61	9.9%	99.9*	1	0.817	651
7.61	24262	939	939	100.0%	15.2%	15.4%	24262	20.18	15.5%	99.9*	-5	0.801	768
6.95	25810	1033	1033	100.0%	25.9%	25.8%	25810						

13.33	26.4%	99.6*	-5	0.770	858		
6.43	27070	1118	1118	100.0%	35.6%	35.5%	27070
10.44	36.4%	99.2*	-2	0.769	942		
6.01	31056	1189	1189	100.0%	50.2%	50.2%	31056
8.28	51.2%	98.4*	-2	0.789	1012		
5.67	33163	1229	1230	99.9%	54.6%	54.1%	33163
8.03	55.6%	98.4*	-4	0.759	1063		
5.38	36384	1336	1336	100.0%	59.9%	59.7%	36384
7.50	61.1%	98.1*	2	0.781	1158		
5.13	38512	1394	1394	100.0%	72.2%	72.0%	38512
6.25	73.5%	97.1*	2	0.782	1221		
4.91	36064	1409	1409	100.0%	74.1%	74.5%	36064
5.79	75.6%	95.8*	-1	0.750	1243		
4.72	40909	1536	1537	99.9%	80.1%	80.6%	40909
5.54	81.7%	95.6*	-1	0.791	1354		
4.55	38488	1530	1530	100.0%	89.2%	89.4%	38488
4.73	91.1%	94.1*	-5	0.730	1364		
4.39	42471	1620	1620	100.0%	106.0%	106.4%	42471
4.23	108.1%	93.5*	-1	0.764	1443		
4.25	44480	1643	1643	100.0%	128.9%	129.0%	44480
3.55	131.3%	91.7*	-2	0.721	1468		
4.13	47223	1740	1740	100.0%	193.5%	193.6%	47223
2.51	197.2%	82.7*	-1	0.708	1567		
4.01	47891	1728	1728	100.0%	261.9%	264.2%	47891
1.79	266.7%	73.1*	-3	0.706	1559		
3.90	50384	1839	1839	100.0%	295.8%	296.9%	50384
1.60	301.3%	63.2*	1	0.707	1659		
3.80	47473	1813	1832	99.0%	341.6%	349.1%	47466
1.25	348.3%	46.8*	-2	0.669	1620		
total	668160	25540	25580	99.8%	44.1%	44.6%	668152
8.36	44.9%	99.9*	-2	0.743	22081		

========== STATISTICS OF INPUT DATA SET ==========

R-FACTORS FOR INTENSITIES OF DATA SET ./XDS_ASCII_osc1.HKL

RESOLUTION LIMIT	R-FACTOR observed	R-FACTOR expected	COMPARED
17.01	8.7%	8.6%	2900

12.03	5.5%	6.5%	6184
9.82	6.6%	7.2%	8509
8.51	9.1%	9.2%	10549
7.61	14.1%	14.2%	12203
6.95	24.1%	24.0%	12865
6.43	33.1%	32.8%	13502
6.01	46.4%	47.0%	15456
5.67	51.6%	50.8%	16542
5.38	57.1%	57.2%	18266
5.13	68.8%	69.0%	19256
4.91	71.5%	72.5%	18014
4.72	77.8%	78.3%	20492
4.55	85.8%	85.9%	19125
4.39	103.3%	104.0%	21233
4.25	126.3%	126.4%	22222
4.13	186.8%	186.0%	23630
4.01	251.3%	255.9%	23859
3.90	289.7%	291.3%	25249
3.80	343.4%	353.1%	23409
total	42.7%	43.2%	333465

R-FACTORS FOR INTENSITIES OF DATA SET ./XDS_ASCII_osc2.HKL

RESOLUTION LIMIT	R-FACTOR observed	R-FACTOR expected	COMPARED
17.01	7.9%	8.5%	2995
12.03	6.2%	7.4%	6210
9.82	7.6%	8.4%	8576
8.51	10.3%	10.8%	10596
7.61	16.3%	16.6%	12059
6.95	27.6%	27.6%	12945
6.43	38.1%	38.2%	13568
6.01	54.0%	53.5%	15600
5.67	57.5%	57.4%	16621
5.38	62.7%	62.1%	18118
5.13	75.6%	75.0%	19256
4.91	76.7%	76.6%	18050
4.72	82.5%	82.9%	20417
4.55	92.7%	92.8%	19363
4.39	108.6%	108.9%	21238
4.25	131.4%	131.5%	22258

4.13	200.5%	201.5%	23593
4.01	272.9%	272.9%	24032
3.90	302.2%	302.8%	25135
3.80	339.9%	345.2%	24057
total	45.4%	45.9%	334687

****
WILSON STATISTICS OF SCALED DATA SET: fae-ip.ahkl
****

Data is divided into resolution shells and a straight line
A - 2*B*SS is fitted to log<I>, where
RES = mean resolution (Angstrom) in shell
SS = mean of (sin(THETA)/LAMBDA)**2 in shell
<I> = mean reflection intensity in shell
BO = (A - log<I>)/(2*SS)
# = number of reflections in resolution shell

WILSON LINE (using all data) : A= 14.823 B= 113.358 CORRELATION= 0.94

#	RES	SS	<I>	log(<I>)	BO
425	20.119	0.001	4.4037E+06	15.298	-384.3
616	12.985	0.001	3.6518E+06	15.111	-97.0
794	10.258	0.002	2.6958E+06	14.807	3.4
909	8.726	0.003	1.5736E+06	14.269	84.4
1008	7.728	0.004	8.3706E+05	13.638	141.6
1101	7.011	0.005	4.4634E+05	13.009	178.4
1184	6.465	0.006	3.7328E+05	12.830	166.6
1276	6.026	0.007	3.1139E+05	12.649	157.9
1355	5.664	0.008	3.0552E+05	12.630	140.7
1401	5.363	0.009	2.4905E+05	12.425	137.9
1509	5.105	0.010	2.5210E+05	12.438	124.3
1562	4.877	0.011	2.3605E+05	12.372	116.6
1584	4.682	0.011	2.2984E+05	12.345	108.6
1680	4.507	0.012	2.0449E+05	12.228	105.4
1707	4.351	0.013	1.8712E+05	12.139	101.6
1805	4.209	0.014	1.3472E+05	11.811	106.7
1818	4.080	0.015	9.7332E+04	11.486	111.1
1913	3.963	0.016	8.9050E+04	11.397	107.6
1893	3.856	0.017	8.0049E+04	11.290	105.0

HIGHER ORDER MOMENTS OF WILSON DISTRIBUTION OF CENTRIC DATA
AS COMPARED WITH THEORETICAL VALUES. (EXPECTED: 1.00)

#	RES	<I**2>/	<I**3>/	<I**4>/
		3<I>**2	15<I>**3	105<I>**4
181	20.119	3.348	12.907	48.763
170	12.985	1.161	1.532	1.789
189	10.258	1.509	3.058	6.404
176	8.726	2.133	4.169	7.041
185	7.728	1.193	2.664	5.895
176	7.011	2.011	4.604	10.170
178	6.465	1.260	1.837	2.841
185	6.026	1.062	1.107	0.992
178	5.664	1.695	2.309	2.803
184	5.363	2.002	5.487	13.731
184	5.105	1.665	2.960	4.928
187	4.877	0.846	0.751	0.617
169	4.682	0.862	0.711	0.549
189	4.507	0.864	0.854	0.773
173	4.351	2.263	6.240	18.430
188	4.209	2.130	4.619	9.766
178	4.080	1.879	2.710	3.979
189	3.963	2.272	3.887	6.398
168	3.856	1.832	1.708	1.496
3427	overall	1.683	3.380	7.770

HIGHER ORDER MOMENTS OF WILSON DISTRIBUTION OF ACENTRIC DATA
AS COMPARED WITH THEORETICAL VALUES. (EXPECTED: 1.00)

#	RES	<I**2>/	<I**3>/	<I**4>/
		2<I>**2	6<I>**3	24<I>**4
244	20.119	1.403	2.636	5.289
446	12.985	1.441	2.144	3.489
605	10.258	1.723	4.085	11.652
733	8.726	1.841	4.616	11.792
823	7.728	1.858	4.185	9.373
925	7.011	1.428	2.688	6.106
1006	6.465	1.568	3.601	9.869
1091	6.026	1.437	2.501	4.544
1177	5.664	1.258	1.946	3.424
1217	5.363	1.342	2.045	3.148

1325	5.105	1.282	1.829	2.676
1375	4.877	1.386	2.325	4.413
1415	4.682	1.236	1.517	1.811
1491	4.507	1.613	3.620	9.675
1534	4.351	1.225	1.851	3.403
1617	4.209	1.393	2.297	4.424
1640	4.080	1.522	2.519	4.742
1724	3.963	1.399	1.860	2.684
1725	3.856	1.518	1.778	2.466
22113	overall	1.443	2.471	4.987

======= CUMULATIVE INTENSITY DISTRIBUTION =======

DEFINITIONS:

<I> = mean reflection intensity

Na(Z)exp = expected number of acentric reflections with I <= Z*<I>

Na(Z)obs = observed number of acentric reflections with I <= Z*<I>

Nc(Z)exp = expected number of centric reflections with I <= Z*<I>

Nc(Z)obs = observed number of centric reflections with I <= Z*<I>

Nc(Z)obs/Nc(Z)exp versus resolution and Z (0.1-1.0)

#	RES	0.1	0.2	0.3	0.4	0.5	0.6	0.7	0.8	0.9	1.0
181	20.119	2.00	1.63	1.49	1.39	1.26	1.20	1.16	1.13	1.09	1.09
170	12.985	1.75	1.57	1.37	1.29	1.28	1.24	1.21	1.16	1.13	1.12
189	10.258	1.41	1.44	1.41	1.35	1.29	1.25	1.19	1.17	1.12	1.09
176	8.726	1.40	1.27	1.28	1.33	1.26	1.23	1.20	1.17	1.16	1.13
185	7.728	1.26	1.39	1.32	1.35	1.32	1.25	1.23	1.20	1.16	1.14
176	7.011	1.21	1.30	1.27	1.25	1.19	1.15	1.17	1.18	1.16	1.17
178	6.465	0.90	1.09	1.12	1.14	1.06	1.05	1.05	1.05	1.04	1.06
185	6.026	1.02	1.08	1.08	1.13	1.15	1.15	1.14	1.13	1.10	1.08
178	5.664	0.95	0.99	1.01	1.06	1.11	1.10	1.11	1.09	1.06	1.02
184	5.363	1.07	0.93	1.00	1.11	1.16	1.21	1.21	1.18	1.21	1.17
184	5.105	1.29	1.16	1.12	1.09	1.11	1.13	1.13	1.13	1.13	1.12
187	4.877	1.08	0.94	1.03	1.05	1.09	1.06	1.07	1.05	1.03	1.03
169	4.682	1.45	1.28	1.24	1.20	1.15	1.11	1.07	1.04	1.05	1.06
189	4.507	1.30	1.09	1.08	1.04	1.05	1.07	1.08	1.11	1.11	1.12
173	4.351	1.05	0.90	0.82	0.77	0.85	0.89	0.89	0.88	0.86	0.89
188	4.209	1.18	0.91	0.84	0.83	0.83	0.82	0.86	0.90	0.92	0.97
178	4.080	1.33	1.09	0.96	0.89	0.83	0.84	0.87	0.88	0.93	0.92
189	3.963	1.32	1.07	1.00	0.96	0.90	0.88	0.90	0.92	0.90	0.89

168	3.856	1.13	0.88	0.77	0.72	0.69	0.67	0.68	0.75	0.76	0.78
3427	overall	1.27	1.16	1.12	1.10	1.08	1.07	1.07	1.06	1.05	1.04

Na(Z)obs/Na(Z)exp versus resolution and Z (0.1-1.0)

#	RES	0.1	0.2	0.3	0.4	0.5	0.6	0.7	0.8	0.9	1.0
244	20.119	3.01	2.22	1.71	1.58	1.48	1.37	1.28	1.24	1.19	1.17
446	12.985	1.77	1.65	1.52	1.39	1.28	1.21	1.14	1.09	1.07	1.03
605	10.258	1.74	1.59	1.45	1.40	1.33	1.27	1.22	1.16	1.12	1.10
733	8.726	1.63	1.70	1.54	1.47	1.44	1.33	1.28	1.22	1.19	1.17
823	7.728	1.34	1.49	1.39	1.33	1.25	1.18	1.16	1.14	1.11	1.08
925	7.011	1.24	1.29	1.30	1.27	1.25	1.17	1.15	1.13	1.09	1.08
1006	6.465	1.43	1.24	1.23	1.22	1.19	1.17	1.15	1.11	1.10	1.08
1091	6.026	1.18	1.11	1.13	1.16	1.14	1.14	1.13	1.13	1.12	1.10
1177	5.664	1.37	1.25	1.20	1.21	1.19	1.16	1.13	1.13	1.11	1.09
1217	5.363	1.36	1.21	1.15	1.15	1.14	1.14	1.14	1.10	1.08	1.06
1325	5.105	1.48	1.12	1.08	1.12	1.11	1.10	1.09	1.09	1.08	1.07
1375	4.877	1.39	1.20	1.15	1.13	1.12	1.11	1.09	1.08	1.06	1.05
1415	4.682	1.44	1.11	1.08	1.11	1.07	1.07	1.06	1.06	1.06	1.05
1491	4.507	1.65	1.23	1.14	1.10	1.11	1.11	1.08	1.06	1.05	1.06
1534	4.351	1.74	1.26	1.11	1.08	1.06	1.04	1.05	1.05	1.05	1.04
1617	4.209	1.99	1.31	1.13	1.12	1.09	1.07	1.05	1.05	1.04	1.04
1640	4.080	2.36	1.50	1.23	1.10	1.03	0.98	0.96	0.95	0.94	0.95
1724	3.963	2.35	1.46	1.16	1.04	0.98	0.97	0.96	0.95	0.94	0.94
1725	3.856	2.66	1.54	1.23	1.09	1.01	0.96	0.93	0.92	0.91	0.91
22113	overall	1.75	1.33	1.20	1.16	1.13	1.10	1.08	1.06	1.05	1.04

List of 52 reflections *NOT* obeying Wilson distribution (Z> 10.0)

h	k	l	RES	Z	Intensity	Sigma	
1	6	4	10.78	18.15	0.4893E+08	0.8271E+06	"alien"
1	10	5	6.62	17.96	0.6704E+07	0.1210E+06	"alien"
40	11	1	4.57	17.56	0.3590E+07	0.7215E+05	"alien"
7	8	10	7.11	16.67	0.7443E+07	0.1131E+06	"alien"
2	7	7	8.70	15.36	0.2418E+08	0.3856E+06	"alien"
1	10	4	6.69	15.32	0.5717E+07	0.1014E+06	"alien"
42	11	1	4.46	15.19	0.3107E+07	0.7240E+05	"alien"
3	15	11	4.27	15.13	0.2038E+07	0.5687E+05	"alien"
7	15	8	4.37	14.78	0.2765E+07	0.5851E+05	"alien"

40	11	2	4.56	14.75	0.3016E+07	0.6351E+05	"alien"
5	8	3	8.29	13.84	0.2177E+08	0.3601E+06	"alien"
43	8	7	4.89	13.80	0.3257E+07	0.6999E+05	"alien"
4	11	10	5.63	13.39	0.4090E+07	0.6890E+05	"alien"
16	7	7	7.75	13.12	0.1098E+08	0.1671E+06	"alien"
17	15	12	4.08	13.01	0.1266E+07	0.5483E+05	"alien"
1	8	5	8.14	12.92	0.1082E+08	0.1637E+06	"alien"
6	0	0	44.97	12.68	0.2686E+09	0.1054E+08	"alien"
4	11	6	5.96	12.59	0.3921E+07	0.6847E+05	"alien"
60	2	1	4.46	12.53	0.2563E+07	0.7388E+05	"alien"
59	3	1	4.48	12.29	0.2513E+07	0.7684E+05	"alien"
12	16	8	4.07	12.23	0.1191E+07	0.4360E+05	"alien"
18	7	4	7.95	12.19	0.1020E+08	0.1605E+06	"alien"
1	15	11	4.27	11.93	0.1608E+07	0.5271E+05	"alien"
13	1	1	19.65	11.91	0.5246E+08	0.9004E+06	"alien"
14	11	4	5.82	11.67	0.3564E+07	0.6255E+05	"alien"
15	6	5	9.07	11.65	0.1833E+08	0.2890E+06	"alien"
2	16	5	4.21	11.65	0.1569E+07	0.5590E+05	"alien"
12	7	3	8.78	11.65	0.1833E+08	0.2913E+06	"alien"
10	2	9	12.31	11.58	0.4227E+08	0.7513E+06	"alien"
47	8	12	4.39	11.35	0.2123E+07	0.6647E+05	"alien"
13	1	27	4.89	11.31	0.2670E+07	0.9373E+05	"alien"
12	14	9	4.55	11.31	0.2312E+07	0.5430E+05	"alien"
3	6	1	11.25	11.21	0.3021E+08	0.5084E+06	"alien"
2	7	4	9.35	11.15	0.1755E+08	0.2782E+06	"alien"
32	9	15	4.79	11.05	0.2609E+07	0.6413E+05	"alien"
17	15	0	4.38	11.03	0.4447E+07	0.1107E+06	"alien"
26	11	6	5.19	11.03	0.2780E+07	0.6220E+05	"alien"
8	7	9	7.97	11.02	0.9223E+07	0.1475E+06	"alien"
10	14	11	4.48	10.76	0.2200E+07	0.5745E+05	"alien"
37	13	9	4.10	10.76	0.1047E+07	0.5465E+05	"alien"
9	17	3	3.97	10.69	0.9517E+06	0.5313E+05	"alien"
1	15	15	4.07	10.63	0.1034E+07	0.5063E+05	"alien"
4	7	4	9.29	10.60	0.1669E+08	0.2777E+06	"alien"
5	15	16	4.00	10.55	0.9394E+06	0.5092E+05	"alien"
19	10	1	6.15	10.49	0.3266E+07	0.6104E+05	"alien"
1	6	1	11.33	10.39	0.2801E+08	0.4679E+06	"alien"
48	7	2	4.86	10.33	0.2437E+07	0.5944E+05	"alien"
65	1	4	4.11	10.25	0.9979E+06	0.6485E+05	"alien"
48	0	5	5.51	10.25	0.5229E+07	0.1254E+06	"alien"
39	6	9	5.51	10.19	0.2538E+07	0.6298E+05	"alien"
20	7	1	7.89	10.17	0.8511E+07	0.1268E+06	"alien"

33 13 4 4.38 10.08 0.1887E+07 0.5782E+05 "alien"

List of 52 reflections *NOT* obeying Wilson distribution (sorted by resolution)
Ice rings could occur at (Angstrom):
3.897,3.669,3.441, 2.671,2.249,2.072, 1.948,1.918,1.883,1.721

h	k	l	RES	Z	Intensity	Sigma
9	17	3	3.97	10.69	0.9517E+06	0.5313E+05
5	15	16	4.00	10.55	0.9394E+06	0.5092E+05
1	15	15	4.07	10.63	0.1034E+07	0.5063E+05
12	16	8	4.07	12.23	0.1191E+07	0.4360E+05
17	15	12	4.08	13.01	0.1266E+07	0.5483E+05
37	13	9	4.10	10.76	0.1047E+07	0.5465E+05
65	1	4	4.11	10.25	0.9979E+06	0.6485E+05
2	16	5	4.21	11.65	0.1569E+07	0.5590E+05
3	15	11	4.27	15.13	0.2038E+07	0.5687E+05
1	15	11	4.27	11.93	0.1608E+07	0.5271E+05
7	15	8	4.37	14.78	0.2765E+07	0.5851E+05
17	15	0	4.38	11.03	0.4447E+07	0.1107E+06
33	13	4	4.38	10.08	0.1887E+07	0.5782E+05
47	8	12	4.39	11.35	0.2123E+07	0.6647E+05
60	2	1	4.46	12.53	0.2563E+07	0.7388E+05
42	11	1	4.46	15.19	0.3107E+07	0.7240E+05
10	14	11	4.48	10.76	0.2200E+07	0.5745E+05
59	3	1	4.48	12.29	0.2513E+07	0.7684E+05
12	14	9	4.55	11.31	0.2312E+07	0.5430E+05
40	11	2	4.56	14.75	0.3016E+07	0.6351E+05
40	11	1	4.57	17.56	0.3590E+07	0.7215E+05
32	9	15	4.79	11.05	0.2609E+07	0.6413E+05
48	7	2	4.86	10.33	0.2437E+07	0.5944E+05
13	1	27	4.89	11.31	0.2670E+07	0.9373E+05
43	8	7	4.89	13.80	0.3257E+07	0.6999E+05
26	11	6	5.19	11.03	0.2780E+07	0.6220E+05
48	0	5	5.51	10.25	0.5229E+07	0.1254E+06
39	6	9	5.51	10.19	0.2538E+07	0.6298E+05
4	11	10	5.63	13.39	0.4090E+07	0.6890E+05
14	11	4	5.82	11.67	0.3564E+07	0.6255E+05
4	11	6	5.96	12.59	0.3921E+07	0.6847E+05
19	10	1	6.15	10.49	0.3266E+07	0.6104E+05
1	10	5	6.62	17.96	0.6704E+07	0.1210E+06

1	10	4	6.69	15.32	0.5717E+07	0.1014E+06
7	8	10	7.11	16.67	0.7443E+07	0.1131E+06
16	7	7	7.75	13.12	0.1098E+08	0.1671E+06
20	7	1	7.89	10.17	0.8511E+07	0.1268E+06
18	7	4	7.95	12.19	0.1020E+08	0.1605E+06
8	7	9	7.97	11.02	0.9223E+07	0.1475E+06
1	8	5	8.14	12.92	0.1082E+08	0.1637E+06
5	8	3	8.29	13.84	0.2177E+08	0.3601E+06
2	7	7	8.70	15.36	0.2418E+08	0.3856E+06
12	7	3	8.78	11.65	0.1833E+08	0.2913E+06
15	6	5	9.07	11.65	0.1833E+08	0.2890E+06
4	7	4	9.29	10.60	0.1669E+08	0.2777E+06
2	7	4	9.35	11.15	0.1755E+08	0.2782E+06
1	6	4	10.78	18.15	0.4893E+08	0.8271E+06
3	6	1	11.25	11.21	0.3021E+08	0.5084E+06
1	6	1	11.33	10.39	0.2801E+08	0.4679E+06
10	2	9	12.31	11.58	0.4227E+08	0.7513E+06
13	1	1	19.65	11.91	0.5246E+08	0.9004E+06
6	0	0	44.97	12.68	0.2686E+09	0.1054E+08

cpu time used by XSCALE 20.9 sec
elapsed wall-clock time 21.2 sec
